# A propensity score approach and a partitioned approach for the self-controlled case series design to evaluate safety of a 2-dose vaccine series: application to myocarditis/pericarditis following mRNA COVID-19 vaccination

**DOI:** 10.1093/aje/kwae141

**Published:** 2024-06-21

**Authors:** Stanley Xu, Lina S Sy, Bing Han, Vennis Hong, Katia J Bruxvoort, Bruno Lewin, Kimberly J Holmquist, Lei Qian

**Affiliations:** Department of Research and Evaluation, Kaiser Permanente Southern California, 100 S. Los Robles Ave, 5th Floor, Pasadena, CA 91101, United States; Department of Health Systems Science, Kaiser Permanente Bernard J. Tyson School of Medicine, Pasadena, CA 91101, United States; Department of Research and Evaluation, Kaiser Permanente Southern California, 100 S. Los Robles Ave, 5th Floor, Pasadena, CA 91101, United States; Department of Research and Evaluation, Kaiser Permanente Southern California, 100 S. Los Robles Ave, 5th Floor, Pasadena, CA 91101, United States; Department of Research and Evaluation, Kaiser Permanente Southern California, 100 S. Los Robles Ave, 5th Floor, Pasadena, CA 91101, United States; Department of Research and Evaluation, Kaiser Permanente Southern California, 100 S. Los Robles Ave, 5th Floor, Pasadena, CA 91101, United States; School of Public Health, University of Alabama at Birmingham, Birmingham, AL 35294, United States; Department of Research and Evaluation, Kaiser Permanente Southern California, 100 S. Los Robles Ave, 5th Floor, Pasadena, CA 91101, United States; Department of Clinical Science, Kaiser Permanente Bernard J. Tyson School of Medicine, Pasadena, CA 91101, United States; Department of Research and Evaluation, Kaiser Permanente Southern California, 100 S. Los Robles Ave, 5th Floor, Pasadena, CA 91101, United States; Department of Research and Evaluation, Kaiser Permanente Southern California, 100 S. Los Robles Ave, 5th Floor, Pasadena, CA 91101, United States

**Keywords:** self-controlled case series, propensity score, simulation, vaccine safety study, myocarditis/pericarditis, COVID-19 mRNA vaccines

## Abstract

The assumption that serious adverse events (SAEs) do not affect subsequent exposure might not hold when evaluating 2-dose vaccine safety through a self-controlled case series (SCCS) design. To address this, we developed: (1) propensity score SCCS (PS-SCCS) using a propensity score model involving SAEs during the risk interval after dose 1 (${R}_1$), and (2) partitioned SCCS (P-SCCS) estimating relative incidence (RI) separately for doses 1 and 2. In simulations, both provided unbiased RIs. Conversely, standard SCCS overestimated RI after dose 2. We applied these approaches to assess myocarditis/pericarditis risks after 2-dose mRNA coronavirus disease 2019 (COVID-19) vaccination in 12- to 39-year-olds. For BNT162b2, PS-SCCS yielded RIs of 1.85 (95% CI, 0.75-4.59) and 11.05 (95% CI, 6.53-18.68) 14 days after doses 1 and 2 respectively; standard SCCS provided similar RI after dose 1 and RI of 12.92 (95% CI, 7.56-22.09) after dose 2. For mRNA-1273, standard SCCS showed RIs of 1.96 (95% CI, 0.56-6.91) after dose 1 and 7.87 (95% CI, 3.33-18.57) after dose 2. As no mRNA-1273 recipients with SAEs during ${R}_1$ received dose 2, P-SCCS was used, yielding similar RI after dose 1 and RI of 6.48 (95% CI, 2.83-14.83) after dose 2. mRNA vaccines were associated with elevated myocarditis/pericarditis risks following dose 2 in 12- to 39-year-olds.

## Introduction

Since December 2020, two mRNA coronavirus disease 2019 (COVID-19) vaccines (BNT162b2 (Pfizer-BioNTech) and mRNA-1273 (Moderna)) have been widely used in the United States.^1^ The two mRNA vaccines were initially authorized as a 2-dose primary series. Post–emergency use authorization observational studies showed associations with some rare, clinically serious adverse events (SAEs) such as myocarditis or pericarditis following mRNA COVID-19 vaccination.^2^^-^^6^ Patone et al^6^ used a self-controlled case series (SCCS) study design while others used a cohort study design or compared postvaccination risk intervals with comparison intervals. However, these studies did not consider the influence of experiencing SAEs after dose 1 of mRNA COVID-19 vaccine on receiving dose 2.

The standard SCCS approach has been widely used for vaccine safety studies.^7^^-^^14^ SCCS is a case-only method in which an individual’s observation period is partitioned into risk and control intervals, and incidence rates of SAEs are compared between the risk and control intervals within individuals, producing relative incidence (RI); thus, SCCS implicitly controls for time-invariant confounders such as sex and race/ethnicity. The analytical data for an SCCS design include the start and end of observation period, date of vaccination, dose indicator, date of SAE, and time-varying covariates such as seasonality. The SCCS design has advantages in vaccine safety studies when those who receive vaccines (vaccinees) and those who do not (unvaccinated comparators) differ greatly in confounders, or when vaccine coverage is high in a population, and traditional cohort and matched cohort designs are not feasible due to a limited number of unvaccinated comparators.^15^

A key assumption of the standard SCCS is that the probability of exposure is not affected by the occurrence of SAEs. In the context of mRNA COVID-19 vaccine safety, SAEs such as myocarditis/pericarditis after receiving dose 1 (vaccine-associated SAE) could decrease the likelihood of receiving dose 2. It has been shown that, if the prior adverse event precludes or reduces the frequency of subsequent exposures, RI will be biased upward if a prevaccination control window is included in the analysis. On the other hand, if the prior adverse event precipitates or increases the frequency of subsequent exposures, RI will be biased downward toward zero.^16^ In this study, we only consider the former because experiencing myocarditis/pericarditis after receiving dose 1 (vaccine-associated SAE) could only decrease the likelihood of receiving dose 2. Although a modified SCCS for censored, perturbed, or curtailed postevent exposures was developed and can handle the influence of a prior SAE on receiving dose 1, it has not been widely used because of its unconventional approach, including a complex estimation approach derived from a pseudolikelihood in a counterfactual framework.^17^ In addition, the existing approach does not account for the influence of vaccine-associated SAEs occurring during the risk interval after dose 1 on receiving dose 2, and it only applies to rare nonrecurrent events and does not allow for recurrent events.^17^ In this study, we propose two approaches to account for the influence of vaccine-associated SAEs on subsequent exposure and demonstrate how they can mitigate potential biases: a propensity score approach for SCCS design (PS-SCCS) and a partitioned SCCS design (P-SCCS). These new approaches accommodate recurrent events. Because we focus on the influence of vaccine-associated SAEs on subsequent exposure, all subjects in the SCCS analytical dataset received at least one dose of a 2-dose vaccine primary series.

## Methods

### Motivation to develop methodology for assessing myocarditis/pericarditis risk after mRNA COVID-19 vaccination

In evaluating myocarditis/pericarditis risks following 2-dose primary series of COVID-19 mRNA vaccination, few studies considered the influence of myocarditis/pericarditis after dose 1 on receiving dose 2. In our study, we identified cases of myocarditis/pericarditis without history of SARS-CoV-2 infection among individuals aged 12-39 years who received at least 1 dose of COVID-19 mRNA vaccine between December 14, 2020, and June 30, 2022, within Kaiser Permanente Southern California. The history of SARS-CoV-2 infection was confirmed by a positive laboratory test or a COVID-19 diagnosis. Our observation period was 90 days before and 90 days after dose 1. We validated incident cases of myocarditis/pericarditis detected in emergency department or inpatient settings through review of medical records. We excluded false cases due to several reasons including but not limited to coding errors, nonincident events, incomplete documentation, and changes in diagnosis. We performed separate analyses for BNT162b2 and mRNA-1273 vaccines, with a 14-day prespecified risk interval for doses 1 and 2. During our investigation, we observed: (1) among BNT162b2 recipients, some who experienced myocarditis/pericarditis in the risk interval after dose 1 received dose 2; (2) in contrast, among mRNA-1273 recipients, none of those who experienced myocarditis/pericarditis within the risk interval following dose 1 received dose 2. To account for these differences, we developed two approaches: PS-SCCS and P-SCCS. We conducted rigorous simulations to evaluate their performance and applied them to evaluate myocarditis/pericarditis risks after 2-dose mRNA COVID-19 vaccination.

### Standard SCCS

In standard SCCS, often risk intervals have been predefined, partially informed by prior studies, or by prior hypotheses based on biological understanding of how vaccines work. The control intervals are periods outside of the risk intervals. For an individual, let ${y}_j$ denote the number of SAEs in interval $j$, $j$ =1, …, *J*; let ${\boldsymbol{X}}_j$ represent the row vector of time-varying covariates; and let $\boldsymbol{\mathrm{\theta}}$ represent the column vector of corresponding coefficients. Conditional Poisson regression models have been used to analyze SCCS data.^7^^,^^8^ Assuming a known risk interval and a constant risk, conditioning on the number of SAEs within an individual, the conditional likelihood function for an individual is:


\begin{align*} & \notag L={\left[\frac{C_0\exp \left({\boldsymbol{X}}_{C_0}\boldsymbol{\mathrm{\theta}} \right)}{\begin{array}{c} C_0\exp \left({\boldsymbol{X}}_{C_0}\boldsymbol{\mathrm{\theta}} \right)+{R}_1\exp \left({\boldsymbol{X}}_{R_1}\boldsymbol{\mathrm{\theta}} +{\mathrm{\beta}}_1\right)+{C}_1\exp \left({\boldsymbol{X}}_{C_1}\boldsymbol{\mathrm{\theta}} \right)\\ +{R}_2\exp \left({\boldsymbol{X}}_{R_2}\boldsymbol{\mathrm{\theta}} +{\mathrm{\beta}}_2\right)+{C}_2\exp \left({\boldsymbol{X}}_{C_2}\boldsymbol{\mathrm{\theta}} \right)\end{array}}\right]}^{y_{C_0}}\\ \notag &\qquad{\left[\frac{\ {R}_1\exp \left({\boldsymbol{X}}_{R_1}\boldsymbol{\mathrm{\theta}} +{\mathrm{\beta}}_1\right)}{\begin{array}{c}C_0\exp \left({\boldsymbol{X}}_{C_0}\boldsymbol{\mathrm{\theta}} \right)+{R}_1\exp \left({\boldsymbol{X}}_{R_1}\boldsymbol{\mathrm{\theta}} +{\mathrm{\beta}}_1\right)+{C}_1\exp \left({\boldsymbol{X}}_{C_1}\boldsymbol{\mathrm{\theta}} \right)\\ +{R}_2\exp \left({\boldsymbol{X}}_{R_2}\boldsymbol{\mathrm{\theta}} +{\mathrm{\beta}}_2\right)+{C}_2\exp \left({\boldsymbol{X}}_{C_2}\boldsymbol{\mathrm{\theta}} \right)\end{array}}\right]}^{y_{R_1}}\\ &\qquad \notag{\left[\frac{C_1\exp \left({\boldsymbol{X}}_{C_1}\boldsymbol{\mathrm{\theta}} \right)}{\begin{array}{c}C_0\exp \left({\boldsymbol{X}}_{C_0}\boldsymbol{\mathrm{\theta}} \right)+{R}_1\exp \left({\boldsymbol{X}}_{R_1}\boldsymbol{\mathrm{\theta}} +{\mathrm{\beta}}_1\right)+{C}_1\exp \left({\boldsymbol{X}}_{C_1}\boldsymbol{\mathrm{\theta}} \right)\\ +{R}_2\exp \left({\boldsymbol{X}}_{R_2}\boldsymbol{\mathrm{\theta}} +{\mathrm{\beta}}_2\right)+{C}_2\exp \left({\boldsymbol{X}}_{C_2}\boldsymbol{\mathrm{\theta}} \right)\end{array}}\right]}^{y_{C_1}} \end{align*}

\begin{align*} \qquad\notag{\left[\frac{R_2\exp \left({\boldsymbol{X}}_{R_2}\boldsymbol{\mathrm{\theta}} +{\mathrm{\beta}}_2\right)}{\begin{array}{c}C_0\exp \left({\boldsymbol{X}}_{C_0}\boldsymbol{\mathrm{\theta}} \right)+{R}_1\exp \left({\boldsymbol{X}}_{R_1}\boldsymbol{\mathrm{\theta}} +{\mathrm{\beta}}_1\right)+{C}_1\exp \left({\boldsymbol{X}}_{C_1}\boldsymbol{\mathrm{\theta}} \right)\\ +{R}_2\exp \left({\boldsymbol{X}}_{R_2}\boldsymbol{\mathrm{\theta}} +{\mathrm{\beta}}_2\right)+{C}_2\exp \left({\boldsymbol{X}}_{C_2}\boldsymbol{\mathrm{\theta}} \right)\end{array}}\right]}^{y_{R_2}} \end{align*}

**Figure 1:**
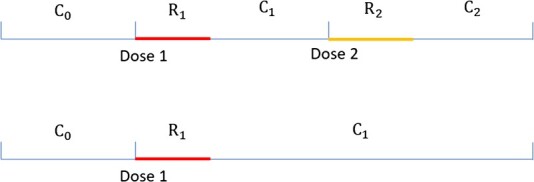
Risk and control intervals for an individual who received a 2-dose primary series of mRNA coronavirus disease 2019 (COVID-19) vaccination and another individual who received only one dose.

(1) \begin{equation*}\qquad{\left[\frac{C_2\exp \left({\boldsymbol{X}}_{C_2}\boldsymbol{\mathrm{\theta}} \right)}{\begin{array}{c} C_0\exp \left({\boldsymbol{X}}_{C_0}\boldsymbol{\mathrm{\theta}} \right)+{R}_1\exp \left({\boldsymbol{X}}_{R_1}\boldsymbol{\mathrm{\theta}} +{\mathrm{\beta}}_1\right)+{C}_1\exp \left({\boldsymbol{X}}_{C_1}\boldsymbol{\mathrm{\theta}} \right)\\ +{R}_2\exp \left({\boldsymbol{X}}_{R_2}\boldsymbol{\mathrm{\theta}} +{\mathrm{\beta}}_2\right)+{C}_2\exp \left({\boldsymbol{X}}_{C_2}\boldsymbol{\mathrm{\theta}} \right)\end{array}}\right]}^{y_{C_2}} \end{equation*}

where ${C}_0$, ${R}_1$, ${C}_1$, ${R}_2$, and ${C}_2$ represent person-time in days for the partitioned intervals (Figure 1); ${y}_{C_0}$, ${y}_{R_1},{y}_{C_1},{y}_{R_2},\textrm{and}\ {y}_{C_2}$are number of SAEs in each of those intervals; for rare SAE, they are binary (1 or 0); ${\mathrm{\beta}}_1$ and ${\mathrm{\beta}}_2$ are the coefficients for the vaccination effects after doses 1 and 2. Maximum likelihood estimates of ${\mathrm{\beta}}_1$ and ${\mathrm{\beta}}_2$ can be obtained by maximizing the likelihood function in equation 1. Estimated RIs after doses 1 and 2 are ${\mathrm{RI}}_1={e}^{\widehat{\beta_1}}$ and ${\mathrm{RI}}_2={e}^{\widehat{\beta_2}}$.

### PS-SCCS considering the influence of adverse events during the risk interval after dose 1 on receiving dose 2 of mRNA COVID-19 vaccination

PS-SCCS uses a propensity score model framework to account for the influence of vaccine-associated SAEs occurring during ${R}_1$ on receiving dose 2 of mRNA COVID-19 vaccine. Partitioned intervals for an individual who received 2-dose primary series of mRNA COVID-19 vaccination are shown in Figure 1, where ${C}_0$, ${R}_1$, ${C}_1$, ${R}_2$, and ${C}_2$ are the control interval before dose 1, the risk interval after dose 1, the control interval after ${R}_1$, the risk interval after dose 2, and the control interval after ${R}_2$, respectively. Note that when one does not receive dose 2, the entire follow-up after ${R}_1$ is ${C}_1$. Let ${V}_2$ denote whether a person received dose 2 within the observation period, and ${z}_{\left({R}_1\right)}$ denote whether any SAE occurred during ${R}_1$. We considered an SAE during ${R}_1$ to be a vaccine-associated event which had an impact on receiving dose 2. A propensity score model can be created among vaccinees, including ${z}_{\left({R}_1\right)}$ as the independent variable: $\mathrm{prob}\ \left({V}_2=1|{z}_{\left({R}_1\right)}\right)=\frac{\exp \left({\mathrm{\pi}}_0+{\mathrm{\pi}}_1{z}_{\left({R}_1\right)}\right)}{1+\exp \left({\mathrm{\pi}}_0+{\mathrm{\pi}}_1{z}_{\left({R}_1\right)}\right)}$, where ${\mathrm{\pi}}_0$ is the intercept, and ${\mathrm{\pi}}_1$ is the corresponding coefficient. With an improved version of the inverse propensity of treatment weights, stabilized weights ($w$), RIs can be obtained directly from the original analytical approach (ie, conditional likelihood) without using a robust variance estimator to account for underestimation of standard error induced by inverse propensity of treatment weights because the original sample size is maintained.^18^^-^^21^

From the propensity score model, we calculated stabilized weights as follows: if ${V}_2=1$, then $w=\frac{\mathrm{prob}\ \left({V}_2=1\right)}{\mathrm{prob}\ \left({V}_2=1|{z}_{\left({R}_1\right)}\right)}$ where $\mathrm{prob}\ \left({V}_2=1\right)$ is the marginal probability of receiving dose 2 in the study population; if ${V}_2=0$, then $w=\frac{1-\mathrm{prob}\ \left({V}_2=1\right)}{1-\mathrm{prob}\ \left({V}_2=1|{z}_{\left({R}_1\right)}\right)}$. We applied stabilized weights to equation 1. The PS-SCCS conditional likelihood function for an individual (Figure 1) is:


(2) \begin{equation*} {L}^W \end{equation*}

Similarly, maximum likelihood estimates of ${\mathrm{\beta}}_1$ and ${\mathrm{\beta}}_2$ can be obtained by maximizing ${L}^W$.

### P-SCCS for estimating RIs after doses 1 and 2

**Figure 2:**
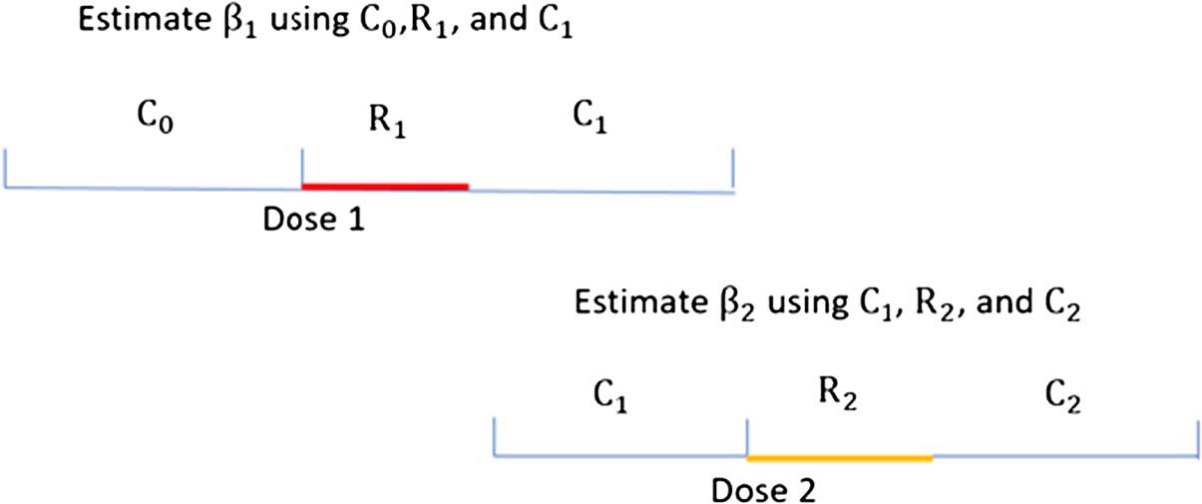
Partitioned self-controlled case series approach (P-SCCS) for estimating relative incidences (RI)s after dose 1 and dose 2 separately.

To account for the impact on receiving dose 2 of experiencing SAEs during ${R}_1$, we also proposed to estimate RIs after doses 1 and 2 separately by partitioning the observation period for two SCCS analyses: We used intervals including ${C}_0$, ${R}_1$, and ${C}_1$ to estimate ${\mathrm{RI}}_1$ and used ${C}_1$, ${R}_2$, and ${C}_2$ to estimate ${\mathrm{RI}}_2$ (Figure 2). Note that information in ${C}_0$ was not used in estimating ${\mathrm{\beta}}_2$ and information in ${C}_2$ was not used in estimating ${\mathrm{\beta}}_1$.The PS-SCCS approach requires a functional propensity score model, wherein at least 1 individual who experienced an SAE during ${R}_1$ after dose 1 received dose 2. In situations where a working PS model was unavailable for certain simulated data, we conducted P-SCCS analyses instead.

### Simulations

#### Simulation algorithm and analyses

Two sets of simulations were performed, one utilizing 7-day risk intervals and the other employing a 14-day risk interval for both doses. Below we provide a detailed description of the simulation utilizing 7-day risk intervals. In each simulated dataset, 10 000 hypothetical individuals who received dose 1 were assumed to have an observation period of 10 weeks ($t=1-70$ days), consisting of 3 control intervals (${C}_0\left[14\ \mathrm{days}\right],{C}_1\left[14\ \mathrm{days}\right],$ and ${C}_2\left[28\ \mathrm{days}\right]$), two risk intervals (${R}_1$[7 days] and ${R}_2$[7 days]) as in Figure 1. We assumed all individuals received dose 1 on day 15, and dose 2 on day 36, if received. For those who did not receive dose 2, there was no ${R}_2$ and ${C}_2$; ${C}_1$ was 49 days.

We first simulated cases, ${y}_t$, during ${C}_0$, ${R}_1$, and ${C}_1$ according to the logit model (3):


(3) \begin{equation*} \mathrm{prob}\ \left({y}_t|{k}_t\right)=\frac{\exp \left({\mathrm{\beta}}_0+{k}_t{\mathrm{\beta}}_1\right)}{1+\exp \left({\mathrm{\beta}}_0+{k}_t{\mathrm{\beta}}_1\right)}, \end{equation*}

where ${y}_t$ is a binary variable for occurrence of SAE, $\mathrm{prob}\ \left({y}_t|{k}_t\right)$ is the probability of ${y}_t$=1 at day $t$, and ${k}_t$ is an indicator variable for the risk interval (${k}_t=1$ for $15\le t\le 21$ and 0 otherwise); ${\mathrm{\beta}}_0$ determines the baseline rate of SAEs during the control intervals, ${C}_0$ and ${C}_1$ (${k}_t$=0); and for rare SAE, approximately, exponentiation of ${\mathrm{\beta}}_1$ is RI for risk interval ${R}_1$. For an individual, if an SAE occurred in ${R}_1$, then ${z}_{(R1)}$=1; otherwise ${z}_{(R1)}$=0.

We then simulated whether an individual received dose 2 according to the logit model (4):


(4) \begin{equation*} {\mathrm{P}}_{V_2}=\mathrm{prob}\ \left({V}_2=1|{z}_{\left({R}_1\right)}\right)=\frac{\exp \left({\mathrm{\pi}}_0+{z}_{\left({R}_1\right)}{\mathrm{\pi}}_1\right)}{1+\exp \left({\mathrm{\pi}}_0+{z}_{\left({R}_1\right)}{\mathrm{\pi}}_1\right)}, \end{equation*}

where ${\mathrm{P}}_{V_2}$ is the probability of receiving dose 2, ${V}_2$ is an indicator for receiving dose 2 (${V}_2=1$), ${\mathrm{\pi}}_0$ is an intercept determining the baseline rate of receiving dose 2, and ${\mathrm{\pi}}_1$ is the coefficient for ${z}_{\left({R}_1\right)}$.

Last, we simulated cases, ${y}_t$, during ${R}_2$ and ${C}_2$ according to the logit model (5):


(5) \begin{align*} \mathrm{prob}\ \left({y}_t|{k}_t\right)=\frac{\exp \left({\mathrm{\beta}}_0+{k}_t{\mathrm{\beta}}_2\right)}{1+\exp \left({\mathrm{\beta}}_0+{k}_t{\mathrm{\beta}}_2\right)}, \end{align*}

where ${k}_t=1$ for $36\le t\le 42$ and ${V}_2=1$, and ${k}_t=0$ for $t>42$ or ${V}_2=0$. For simplicity, we set ${\mathrm{\beta}}_1={\mathrm{\beta}}_2$. Utilizing 7-day risk intervals, we simulated 72 scenarios with three baseline adverse event rates (${\mathrm{\beta}}_0$), two rates of receiving dose 2 (${\mathrm{\pi}}_0$), four impact levels of experiencing SAEs during ${R}_1$ on receiving dose 2 (${\mathrm{\pi}}_1$), and three different log (RI). Three values of ${\mathrm{\beta}}_0$, −9, −8, and −7, were used to represent annual event rates of 4.5%, 12.2%, and 33.3%, respectively. We chose ${\mathrm{\pi}}_0=\log \left(\frac{2}{3}\right)$ and $\log (4)$, representing baseline vaccination rate of dose 2, ${\mathrm{P}}_{V_2}^0=\mathrm{prob}\ \left({V}_2=1|\ {z}_{\left({R}_1\right)}=0\right)$ = 40% and 80%, meaning that 40% and 80% of those who did not experience SAEs during ${R}_1$ received dose 2, respectively. When ${\mathrm{\pi}}_0=\log (4)$ and ${\mathrm{\pi}}_1=0,-2,-3,\textrm{and}-4$, the probability of receiving dose 2 among those who experienced SAEs during ${R}_1$, ${\mathrm{P}}_{V_2}^1=\mathrm{prob}\ \left({V}_2=1|\ {z}_{\left({R}_1\right)}=1\right)$, was 80%, 35.1%, 16.6%, and 6.8%, respectively. Table 1 shows the number of observed cases for two baseline rates of dose 2 vaccination, three ${\mathrm{\beta}}_0$ values, and four ${\mathrm{\pi}}_1$ values under the null hypothesis ${\mathrm{\beta}}_1={\mathrm{\beta}}_2=0$. Under the alternative hypothesis, for simplicity, we set ${\mathrm{RI}}_1={\mathrm{RI}}_2=1.5,2.0,4.0$. In each simulated dataset, the number of SAEs for each of five intervals was calculated by summing ${y}_t$ across intervals within each individual. Because SAEs were rare in our simulation study and the real-world example, ${y}_{C_0}$, ${\mathrm{y}}_{{\mathrm{R}}_1},{y}_{C_1},{y}_{R_2},\textrm{and}\ {y}_{C_2}$were binary outcomes. Each simulated SCCS dataset was analyzed using the three analytical approaches described above. We simulated 1000 replicas for each scenario and the following evaluation metrics were obtained from the 1000 replicas.

**Table 1:** Under the null hypothesis (${\mathrm{\beta}}_1$=${\mathrm{\beta}}_2=0$) and a 7-day risk interval for doses 1 and 2 (${R}_1={R}_2=7\ \textrm{days}$), number of datasets with $S=0$^a^, average number of cases (*n*), and type I error rates by different baseline rate of dose 2 vaccination (${\mathrm{\pi}}_0$), the impact of experiencing SAEs during ${R}_1$ on dose 2 vaccination (${\mathrm{\pi}}_1$), and baseline SAE rate (β_0_) from 1000 simulated datasets with 10 000 individuals in each dataset.

**Simulation parameters**			**Number of datasets with** $\boldsymbol{S}=\mathbf{0}$^**a**^	**Average number of cases (*n*)**			**Type I error rate, %**					
							**Standard SCCS**		**PS-SCCS** ^**e**^		**P-SCCS** ^**f**^	
${\boldsymbol{\Pi}}_{\mathbf{0}}$	${\boldsymbol{\Pi}}_{\mathbf{1}}$	β_0_		***C*** ^**b**^	${\boldsymbol{R}}_{\mathbf{1}}$ ^**c**^	${\boldsymbol{R}}_{\mathbf{2}}$ ^**d**^	${\boldsymbol{R}}_{\mathbf{1}}$ ^**c**^	${\boldsymbol{R}}_{\mathbf{2}}$ ^**d**^	${\boldsymbol{R}}_{\mathbf{1}}$ ^**c**^	${\boldsymbol{R}}_{\mathbf{2}}$ ^**d**^	${\boldsymbol{R}}_{\mathbf{1}}$ ^**c**^	${\boldsymbol{R}}_{\mathbf{2}}$ ^**d**^
$\log \left(\frac{2}{3}\right)$	0	−7	0	550	64	26	5.6	3.6	5.5	3.6	5.3	3.2
		−8	1	202	23	9	5.1	4.5	5.1	4.3	4.8	4.1
		−9	37	74	9	3	4.0	3.0	3.9	2.8	3.7	2.5
	−2	−7	6	550	64	25	5.8	6.5	5.2	3.8	5.3	3.1
		−8	142	202	23	9	5.3	6.3	5.6	4.3	4.8	4.2
		−9	495	74	9	3	4.3	4.6	3.3	3.0	3.7	2.5
	−3	−7	128	550	54	25	6.0	8.1	5.5	4.1	5.3	3.0
		−8	462	202	23	9	5.3	6.6	5.2	4.2	4.8	4.2
		−9	765	74	9	3	4.3	4.7	3.3	2.9	3.7	2.5
	−4	−7	453	549	63	25	6.0	8.8	5.4	3.5	5.3	2.9
		−8	750	202	23	9	5.3	6.8	4.8	4.4	4.8	4.2
		−9	900	74	9	3	4.3	4.8	3.7	2.7	3.7	2.5
$\log (4)$	0	−7	0	524	64	51	5.0	5.1	5.1	5.1	5.3	5.1
		−8	0	193	23	17	4.8	3.8	4.9	4.5	4.8	3.9
		−9	1	71	9	7	4.0	3.7	4.4	3.6	3.7	2.5
	−2	−7	0	524	63	51	6.5	7.1	6.5	5.1	6.1	4.6
		−8	1	193	23	19	4.5	4.9	5.0	4.3	4.3	3.6
		−9	64	71	8	7	5.9	4.3	5.1	4.1	3.5	2.5
	−3	−7	0	524	63	51	6.0	9.3	5.3	5.0	5.3	4.8
		−8	24	193	23	19	4.5	6.9	4.7	4.5	4.4	3.9
		−9	237	71	9	7	4.2	4.5	3.5	3.3	3.7	2.4
	−4	−7	10	523	64	51	6.2	11.6	5.7	5.4	5.3	4.8
		−8	201	193	23	19	4.9	7.3	5.3	4.9	4.8	4.0
		−9	580	71	9	7	4.3	5.2	3.6	2.8	3.7	2.4

Abbreviations: SAE, serious adverse events; SCCS, self-controlled case series.

^a^ $S=\sum{V}_{2i}\mid{y}_{\left({R}_1\right)=1}=0,$ none of those who experienced SAEs during ${R}_1$ received dose 2;

^c^Control intervals included ${C}_0\ \left(14\ \mathrm{days}\right),{C}_1\left(14\ \mathrm{days}\right),\mathrm{and}$  ${C}_2\left(28\ \mathrm{days}\right)$ with a total person-time of 56 days among those who received dose 2, and included ${C}_0\left(14\ \mathrm{days}\right)\ \mathrm{and}$  ${C}_1\left(49\ \mathrm{days}\right)$ with a total person-time of 63 days among those who did not receive dose 2.

^c^The risk interval after dose 1.

^d^The risk interval after dose 2.

^e^Propensity score approach for SCCS design.

^f^Partitioned SCCS approach.

#### Simulation evaluation metrics

1. Type I error rate: The type I error rate was calculated as the proportion of replicated datasets where a statistical test falsely rejected a true null hypothesis (${\mathrm{\beta}}_1={\mathrm{\beta}}_2=0$) at a significance level of 5.0%.
2. Mean of point estimate and average standard deviation: We provided the mean of point estimates and the average standard deviations for the RIs over 1000 replicas.
3. Empirical power: Empirical power was calculated as the proportion of datasets where a statistical test correctly rejected a false null hypothesis when simulations were performed under the true alternative RIs.

All analyses were conducted using SAS Enterprise Guide 8.2 (SAS Institute Inc., Cary, North Carolina). A SAS program is available for fitting the three SCCS models for 2-dose series such as COVID-19 mRNA vaccines (Appendix S1). This study was approved by the Kaiser Permanente Southern California Institutional Review Board.

## Results

### Simulation results

We present results from standard SCCS and P-SCCS for all simulation scenarios. For PS-SCCS results, we used P-SCCS instead of PS-SCCS when in a simulated dataset none of those who experienced SAEs during ${R}_1$ received dose 2 $\left(S=\left[\sum{V}_{2i}|{z}_{\left({R}_1\right)i}=1\right]=0\right)$ and the propensity score model did not work. When ${\mathrm{P}}_{V_2}^0$, ${\mathrm{\pi}}_1$, and ${\mathrm{\beta}}_0$ had lower values, the number of simulated datasets with $S=0$ increased, such that more of the results of PS-SCCS were those from the P-SCCS approach. Simulation results were similar for 7-day risk and 14-day risk intervals. Below, we provided a detailed description of the simulation results for 7-day risk intervals.

#### Type I error rates

Type I error rates are shown in Table 1. When experiencing SAEs during ${R}_1$ had no impact on dose 2 vaccination (${\mathrm{\pi}}_1$=0), all three approaches yielded type I error rates close to the nominal value (5.0%) for ${\mathrm{RI}}_1$and ${\mathrm{RI}}_2$ regardless of the baseline dose 2 vaccination rate (${\mathrm{P}}_{V_2}^0$) and the baseline event rate (${\mathrm{\beta}}_0$). Different values of ${\mathrm{\pi}}_1$ had only a slight impact on the type I error rates for ${\mathrm{RI}}_1$. However, when the impact on dose 2 vaccination of experiencing SAEs during ${R}_1$ increased (${\mathrm{\pi}}_1=-3,-4$), and baseline rates of adverse events were higher (eg, ${\mathrm{\beta}}_0=-7$), the standard SCCS inflated type I error rates up to 11.6% for ${\mathrm{RI}}_2$, while type I error rates of PS-SCCS and P-SCCS remained close to the nominal value.

#### Average number of cases under the alternative hypothesis

Under the alternative hypothesis, the number of cases ranged from 13 to 254 in ${R}_1$ and 5 to 204 in ${R}_2$ (Table 2). The number of simulated datasets with $S=0$ increased when ${\mathrm{P}}_{V_2}^0$, ${\mathrm{\pi}}_1$, ${\mathrm{\beta}}_0$, and ${\mathrm{\beta}}_1$ decreased. When ${\mathrm{P}}_{V_2}^0=40\%$, ${\mathrm{\pi}}_1=-4$, ${\mathrm{\beta}}_0=-9$, and ${e}^{\beta_1}={e}^{\beta_2}=1.5$, 765 out of 1000 datasets had $S=0$ (Table 2).

**Table 2:** Under the alternative hypothesis (${\mathrm{\beta}}_1$ = ${\mathrm{\beta}}_2\ne 0$) and a 7-day risk interval for doses 1 and 2 (${R}_1={R}_2=7\ \mathrm{days}$), number of datasets with $S=0$^a^ and average number of cases by different baseline rate of dose 2 vaccination (${\mathrm{\pi}}_0$), relative incidences (${\mathrm{\beta}}_1$ and ${\mathrm{\beta}}_2$), the impact of experiencing SAEs during ${R}_1$ on dose 2 vaccination (${\mathrm{\pi}}_1$), and baseline SAE rate (β_0_) from 1000 simulated datasets with 10 000 individuals in each dataset.

**Simulation parameters**				**Number of datasets with** $\boldsymbol{S}=\mathbf{0}$^**a**^	**Average number of cases**		
${\boldsymbol{\Pi}}_{\mathbf{0}}$	${\boldsymbol{e}}^{\beta_1}$ **=** ${\boldsymbol{e}}^{\beta_2}$	${\boldsymbol{\Pi}}_{\mathbf{1}}$	β_0_		***C*** ^**b**^	${\boldsymbol{R}}_{\mathbf{1}}$ ^**c**^	${\boldsymbol{R}}_{\mathbf{2}}$ ^**d**^
$\log \left(\frac{2}{3}\right)$	1.50	0	−7	0	550	95	38
			−8	0	202	35	14
			−9	4	74	13	5
		−2	−7	0	550	95	38
			−8	55	202	35	14
			−9	342	74	13	5
		−3	−7	39	550	96	38
			−8	314	202	35	14
			−9	656	74	13	5
		−4	−7	292	550	95	38
			−8	667	202	35	14
			−9	765	74	13	5
	2.00	0	−7	0	550	127	51
			−8	0	202	46	19
			−9	1	73	17	7
		−2	−7	0	550	127	50
			−8	21	202	47	19
			−9	248	74	17	7
		−3	−7	20	550	127	50
			−8	210	202	47	19
			−9	580	74	17	7
		−4	−7	217	550	128	50
			−8	571	202	47	19
			−9	807	74	17	7
	4.00	0	−7	0	550	254	102
			−8	0	202	93	38
			−9	0	74	34	14
		−2	−7	0	550	254	100
			−8	0	202	93	37
			−9	57	74	34	14
		−3	−7	0	550	254	100
			−8	47	202	94	37
			−9	323	74	34	14
		−4	−7	46	550	254	100
			−8	303	202	94	37
			−9	671	74	34	14
$\log (4)$	1.50	0	−7	0	525	95	77
			−8	0	193	35	28
			−9	0	71	13	10
		−2	−7	0	525	95	76
			−8	0	193	35	28
			−9	9	71	13	10
		−3	−7	0	524	95	76
			−8	8	193	35	28
			−9	127	71	13	10
		−4	−7	2	525	95	76
			−8	86	193	35	28
			−9	423	71	13	10
	2.00	0	−7	0	524	126	102
			−8	0	193	47	37
			−9	0	71	17	14
		−2	−7	0	525	127	101
			−8	0	193	47	37
			−9	3	71	17	14
		−3	−7	0	524	127	101
			−8	1	193	47	37
			−9	57	71	17	14
		−4	−7	0	525	127	101
			−8	36	193	47	37
			−9	319	71	17	14
	4.00	0	−7	0	524	254	204
			−8	0	193	94	75
			−9	0	71	34	27
		−2	−7	0	525	254	201
			−8	0	193	94	65
			−9	0	71	34	27
		−3	−7	0	525	254	200
			−8	0	193	94	74
			−9	8	71	34	27
		−4	−7	0	525	254	199
			−8	2	193	94	74
			−9	91	71	34	27

Abbreviation: SAE, serious adverse events.

^a^ $S=\sum{V}_{2i}\mid{y}_{\left({R}_1\right)=1}=0,$ none of those who experienced SAEs during ${R}_1$ received dose 2;

^c^Control intervals included ${C}_0\left(14\ \mathrm{days}\right),{C}_1\left(14\ \mathrm{days}\right),\mathrm{and}$  ${C}_2\left(28\ \mathrm{days}\right)$ with a total person-time of 56 days among those who received dose 2; control intervals included ${C}_0\ \left(14\ \mathrm{days}\right)\ \mathrm{and}$  ${C}_1\left(49\ \mathrm{days}\right)$ with a total person-time of 63 days among those who did not receive dose 2.

^c^The risk interval after dose 1.

^d^The risk interval after dose 2.

#### Mean and standard error of RIs, percent bias, and empirical power under the alternative hypothesis

When experiencing SAEs during ${R}_1$ had no impact on receiving dose 2 (${\mathrm{\pi}}_1=0$), all three approaches produced unbiased estimates of RIs and comparable empirical power (Tables 3 and 4). When ${\mathrm{P}}_{V_2}^0=40\%$, ${\mathrm{\pi}}_1=-4$, ${\mathrm{\beta}}_0=-9$, and ${e}^{\beta_1}={e}^{\beta_2}=1.5$, all three approaches yielded an unbiased estimate of ${e}^{\beta_1}$; however, standard SCCS overestimated ${e}^{{\hat{\mathrm{\beta}}}_2}$ with mean ${e}^{{\hat{\mathrm{\beta}}}_2}=1.86$ (24.0% bias), while PS-SCCS yielded mean ${e}^{{\hat{\mathrm{\beta}}}_2}=1.57$ (4.7% bias) and P-SCCS estimated ${e}^{{\hat{\mathrm{\beta}}}_2}=1.58$ (5.3% bias) (Table 3). Note that because 765 datasets out of 1000 had $\mathrm{S}=0,$ 765 evaluation measures were those from P-SCCS. In most simulated datasets, P-SCCS produced a slightly larger standard deviation of ${e}^{{\hat{\mathrm{\beta}}}_2}$ and slightly lower empirical power than PS-SCCS under all scenarios possibly because P-SCCS did not use ${C}_0$ for estimating ${e}^{{\hat{\mathrm{\beta}}}_2}$. On the other hand, standard SCCS inflated empirical power for ${e}^{{\hat{\mathrm{\beta}}}_2}$ compared with PS-SCCS.

**Table 3:** Under the alternative hypothesis (${\mathrm{\beta}}_1$=${\mathrm{\beta}}_2\ne 0$), a 7-day risk interval for doses 1 and 2 (${R}_1={R}_2=7\ \mathrm{days}$), and baseline rate of dose 2 vaccination = 40%, the estimated RIs (${e}^{{\hat{\mathrm{\beta}}}_1}$and ${e}^{{\hat{\mathrm{\beta}}}_2}$) (standard deviation) and empirical power by relative incidences (${\mathrm{\beta}}_1$ and ${\mathrm{\beta}}_2$), the impact of experiencing SAEs during ${R}_1$ on dose 2 vaccination (${\mathrm{\pi}}_1$), and baseline SAE rate (β_0_) from 1000 simulated datasets with 10 000 individuals for each dataset.

**Simulation parameters**			**Standard SCCS** ^**a**^				**PS-SCCS** ^**b**^				**P-SCCS** ^**c**^			
			${\boldsymbol{e}}^{{\hat{\mathrm{\beta}}}_{\mathbf{1}}}$ **(SD)**	${\boldsymbol{e}}^{{\hat{\mathrm{\beta}}}_{\mathbf{2}}}$ **(SD)**	**Empirical power, %**		${\boldsymbol{e}}^{{\hat{\mathrm{\beta}}}_{\mathbf{1}}}$ **(SD)**	${\boldsymbol{e}}^{{\hat{\mathrm{\beta}}}_{\mathbf{2}}}$ **(SD)**	**Empirical power, %**		${\boldsymbol{e}}^{{\hat{\mathrm{\beta}}}_{\mathbf{1}}}$ **(SD)**	${\boldsymbol{e}}^{{\hat{\mathrm{\beta}}}_{\mathbf{2}}}$ **(SD)**	**Empirical power, %**	
**(** ${\boldsymbol{e}}^{\beta_{\mathbf{1}}}$ **) = (** ${\boldsymbol{e}}^{\beta_{\mathbf{2}}}$ **)**	${\boldsymbol{\Pi}}_{\mathbf{1}}$	${\boldsymbol{\mathrm{\beta}}}_{\mathbf{0}}$	${\boldsymbol{R}}_{\mathbf{1}}$ ^**d**^	${\boldsymbol{R}}_{\mathbf{2}}$ ^**e**^	${\boldsymbol{R}}_{\mathbf{1}}$ ^**d**^	${\boldsymbol{R}}_{\mathbf{2}}$ ^**e**^	${\boldsymbol{R}}_{\mathbf{1}}$ ^**d**^	${\boldsymbol{R}}_{\mathbf{2}}$ ^**e**^	${\boldsymbol{R}}_{\mathbf{1}}$ ^**d**^	${\boldsymbol{R}}_{\mathbf{2}}$ ^**e**^	${\boldsymbol{R}}_{\mathbf{1}}$ ^**d**^	${\boldsymbol{R}}_{\mathbf{2}}$ ^**e**^	${\boldsymbol{R}}_{\mathbf{1}}$ ^**d**^	${\boldsymbol{R}}_{\mathbf{2}}$ ^**e**^
1.50	0	−7	1.49 (0.17)	1.49 (0.25)	94.1	60.5	1.50 (0.17)	1.49 (0.25)	93.5	59.3	1.50 (0.19)	1.50 (0.26)	89.8	56.8
		−8	1.48 (0.27)	1.51 (0.43)	54.0	29.8	1.49 (0.27)	1.51 (0.44)	54.0	30.2	1.49 (0.30)	1.51 (0.45)	47.5	26.6
		−9	1.53 (0.46)	1.55 (0.77)	28.8	16.8	1.53 (0.47)	1.54 (0.76)	29.2	17.0	1.54 (0.51)	1.57 (0.84)	22.9	12.9
	−2	−7	1.50 (0.17)	1.72 (0.30)	94.2	82.7	1.50 (0.16)	1.50 (0.26)	93.7	61.2	1.50 (0.18)	1.50 (0.26)	90.3	58.7
		−8	1.51 (0.29)	1.73 (0.53)	59.1	45.1	1.51 (0.30)	1.51 (0.45)	58.5	30.2	1.51 (0.32)	1.51 (0.46)	50.3	26.6
		−9	1.54 (0.47)	1.79 (0.91)	30.4	23.4	1.54 (0.47)	1.55 (0.79)	28.2	15.0	1.54 (0.51)	1.57 (0.84)	22.9	12.8
	−3	−7	1.51 (0.16)	1.76 (0.31)	95.0	84.7	1.51 (0.18)	1.51 (0.29)	93.7	60.7	1.51 (0.18)	1.51 (0.26)	91.5	58.1
		−8	1.49 (0.28)	1.76 (0.53)	57.1	47.6	1.50 (0.30)	1.51 (0.46)	54.2	28.2	1.49 (0.30)	1.51 (0.46)	48.6	26.0
		−9	1.54 (0.47)	1.83 (0.93)	30.7	25.0	1.54 (0.49)	1.55 (0.81)	25.7	13.3	1.54 (0.50)	1.57 (0.84)	22.5	12.6
	−4	−7	1.51 (0.17)	1.77 (0.31)	94.2	85.6	1.50 (0.17)	1.50 (0.28)	92.2	58.9	1.50 (0.18)	1.50 (0.26)	90.3	57.7
		−8	1.51 (0.28)	1.78 (0.53)	58.1	48.5	1.50 (0.29)	1.50 (0.45)	52.4	26.9	1.50 (0.30)	1.51 (0.46)	50.3	25.9
		−9	1.54 (0.47)	1.86 (0.96)	29.8	25.3	1.54 (0.51)	1.57 (0.81)	23.4	12.6	1.54 (0.51)	1.58 (0.82)	22.6	12.3
2.00	0	−7	2.00 (0.19)	2.00 (0.31)	100.0	98.3	1.99 (0.19)	2.00 (0.31)	100.0	97.1	1.99 (0.21)	2.01 (0.32)	100.0	97.6
		−8	1.98 (0.31)	2.00 (0.51)	97.1	71.5	1.98 (0.31)	2.00 (0.51)	97.0	70.7	1.99 (0.34)	2.01 (0.54)	94.8	67.6
		−9	2.06 (0.54)	2.07 (0.88)	74.4	38.7	2.06 (0.55)	2.06 (0.88)	74.1	38.1	2.09 (0.61)	2.09 (0.97)	66.3	31.7
	−2	−7	2.01 (0.20)	2.38 (0.38)	100.0	99.8	1.99 (0.20)	2.00 (0.32)	100.0	98.1	2.00 (0.22)	2.00 (0.32)	100.0	97.3
		−8	2.01 (0.33)	2.38 (0.63)	96.9	83.9	2.00 (0.33)	2.00 (0.51)	96.8	70.5	2.00 (0.36)	2.00 (0.54)	94.5	67.5
		−9	2.05 (0.55)	2.46 (1.11)	72.2	50.4	2.04 (0.56)	2.05 (0.90)	69.3	37.1	2.04 (0.60)	2.07 (0.95)	63.0	32.0
	−3	−7	2.01 (0.20)	2.46 (0.39)	100.0	99.8	1.99 (0.20)	2.01 (0.35)	100.0	98.2	2.00 (0.22)	2.00 (0.32)	100.0	97.3
		−8	2.01 (0.33)	2.46 (0.66)	96.9	85.5	1.99 (0.33)	2.00 (0.52)	96.2	70.0	2.00 (0.36)	2.00 (0.54)	94.5	67.4
		−9	2.05 (0.56)	2.56 (1.15)	71.2	52.3	2.04 (0.59)	2.08 (0.95)	65.2	34.9	2.05 (0.61)	2.09 (0.98)	61.0	33.0
	−4	−7	2.02 (0.20)	2.48 (0.40)	100.0	99.8	2.00 (0.22)	2.00 (0.37)	100.0	97.8	2.00 (0.22)	2.00 (0.32)	100.0	97.2
		−8	2.01 (0.33)	2.48 (0.67)	97.0	85.8	1.99 (0.35)	1.99 (0.53)	95.3	68.5	2.00 (0.36)	2.01 (0.54)	94.5	67.0
		−9	2.06 (0.56)	2.59 (1.17)	72.1	52.6	2.06 (0.60)	2.08 (0.96)	63.6	33.4	2.06 (0.61)	2.09 (0.97)	62.1	32.4
4.00	0	−7	3.98 (0.29)	4.00 (0.48)	100.0	100.0	3.98 (0.29)	4.00 (0.48)	100.0	100.0	3.98 (0.34)	4.01 (0.51)	100.0	100.0
		−8	3.98 (0.50)	4.02 (0.76)	100.0	100.0	3.98 (0.50)	4.03 (0.75)	100.0	100.0	4.00 (0.58)	4.04 (0.84)	100.0	100.0
		−9	4.03 (0.82)	4.10 (1.31)	100.0	95.4	4.03 (0.83)	4.10 (1.29)	100.0	95.1	4.08 (0.95)	4.19 (1.53)	99.6	94.1
	−2	−7	4.04 (0.31)	5.46 (0.71)	100.0	100.0	3.99 (0.31)	4.00 (0.50)	100.0	100.0	3.99 (0.36)	4.01 (0.52)	100.0	100.0
		−8	4.04 (0.50)	5.50 (1.14)	100.0	100.0	4.00 (051)	4.05 (0.83)	100.0	100.0	4.02 (0.58)	4.04 (0.85)	100.0	100.0
		−9	4.06 (0.83)	5.67 (2.04)	100.0	98.5	4.02 (0.83)	4.10 (1.41)	99.9	95.3	4.06 (0.97)	4.18 (1.02)	99.6	93.5
	−3	−7	4.04 (0.31)	5.80 (0.77)	100.0	100.0	3.99 (0.31)	4.01 (0.54)	100.0	100.0	3.99 (0.36)	4.01 (0.52)	100.0	100.0
		−8	4.05 (0.51)	5.85 (1.24)	100.0	100.0	3.99 (0.51)	4.02 (0.82)	100.0	100.0	4.01 (0.59)	4.04 (0.85)	100.0	100.0
		−9	4.09 (0.85)	6.02 (2.18)	100.0	98.9	4.05 (0.87)	4.11 (1.39)	99.7	94.9	4.07 (0.97)	4.17 (1.52)	99.6	93.2
	−4	−7	4.05 (0.31)	5.95 (0.80)	100.0	100.0	3.99 (0.34)	4.03 (0.67)	100.0	100.0	3.99 (0.36)	4.01 (0.52)	100.0	100.0
		−8	4.05 (0.51)	5.99 (1.28)	100.0	100.0	4.00 (0.53)	4.01 (0.83)	100.0	100.0	4.01 (0.59)	4.04 (0.85)	100.0	100.0
		−9	4.09 (0.85)	6.18 (2.24)	100.0	99.0	4.06 (0.91)	4.12 (1.44)	99.7	93.8	4.07 (0.97)	4.17 (1.51)	99.6	93.2

Abbreviations: SAE, serious adverse events; SCCS, self-controlled case series; SD, standard deviation.

^a^Control intervals included ${C}_0\left(14\ \mathrm{days}\right),{C}_1\left(14\ \mathrm{days}\right),\mathrm{and}$  ${C}_2\left(28\ \mathrm{days}\right)$ with a total person-time of 56 days among those who received dose 2; control intervals included ${C}_0\left(14\ \mathrm{days}\right)\ \mathrm{and}$  ${C}_1\left(49\ \mathrm{days}\right)$ with a total person-time of 63 days among those who did not receive dose 2.

^c^Propensity score approach for SCCS design.

^c^Partitioned SCCS approach.

^d^The risk interval after dose 1.

^e^The risk interval after dose 2.

**Table 4:** Under the alternative hypothesis (${\mathrm{\beta}}_1$ = ${\mathrm{\beta}}_2\ne 0$), a 7-day risk interval for doses 1 and 2 (${R}_1={R}_2=7\ \mathrm{days}$), and baseline rate of dose 2 vaccination = 80%, the estimated RIs (${e}^{{\hat{\mathrm{\beta}}}_1}$and ${e}^{{\hat{\mathrm{\beta}}}_2}$) (standard deviation) and empirical power by relative incidences (${\mathrm{\beta}}_1$ and ${\mathrm{\beta}}_2$), the impact of experiencing SAEs during ${R}_1$ on dose 2 vaccination (${\mathrm{\pi}}_1$), and baseline SAE rate (β_0_) from 1000 simulated datasets with 10 000 individuals for each dataset.

**Simulation parameters**			**Standard SCCS** ^**a**^				**PS-SCCS** ^**b**^				**P-SCCS** ^**c**^			
			${\boldsymbol{e}}^{{\hat{\mathrm{\beta}}}_{\mathbf{1}}}$ **(SD)**	${\boldsymbol{e}}^{{\hat{\mathrm{\beta}}}_{\mathbf{2}}}$ **(SD)**	**Empirical power, %**		${\boldsymbol{e}}^{{\hat{\mathrm{\beta}}}_{\mathbf{1}}}$ **(SD)**	${\boldsymbol{e}}^{{\hat{\mathrm{\beta}}}_{\mathbf{2}}}$ **(SD)**	**Empirical power, %**		${\boldsymbol{e}}^{{\hat{\mathrm{\beta}}}_{\mathbf{1}}}$ **(SD)**	${\boldsymbol{e}}^{{\hat{\mathrm{\beta}}}_{\mathbf{2}}}$ **(SD)**	**Empirical power, %**	
**(** ${\boldsymbol{e}}^{\beta_{\mathbf{1}}}$ **) = (** ${\boldsymbol{e}}^{\beta_{\mathbf{2}}}$ **)**	${\boldsymbol{\Pi}}_{\mathbf{1}}$	${\mathrm{\beta}}_{\mathbf{0}}$	${\boldsymbol{R}}_{\mathbf{1}}$ ^**d**^	${\boldsymbol{R}}_{\mathbf{2}}$ ^**e**^	${\boldsymbol{R}}_{\mathbf{1}}$ ^**d**^	${\boldsymbol{R}}_{\mathbf{2}}$ ^**e**^	${\boldsymbol{R}}_{\mathbf{1}}$ ^**d**^	${\boldsymbol{R}}_{\mathbf{2}}$ ^**e**^	${\boldsymbol{R}}_{\mathbf{1}}$ ^**d**^	${\mathbf{R}}_{\mathbf{2}}$ ^**e**^	${\boldsymbol{R}}_{\mathbf{1}}$ ^**d**^	${\boldsymbol{R}}_{\mathbf{2}}$ ^**e**^	${\boldsymbol{R}}_{\mathbf{1}}$ ^**d**^	${\boldsymbol{R}}_{\mathbf{2}}$ ^**e**^
1.50	0	−7	1.49 (0.17)	1.50 (0.19)	91.7	86.9	1.49 (0.17)	1.50 (0.19)	91.7	86.6	1.50 (0.18)	1.50 (0.19)	90.3	84.8
		−8	1.49 (0.28)	1.50 (0.30)	56.8	47.8	1.49 (0.28)	1.50 (0.30)	56.5	47.5	1.50 (0.30)	1.50 (0.31)	50.3	46.1
		−9	1.52 (0.46)	1.50 (0.50)	28.7	23.1	1.50 (0.48)	1.50 (0.49)	28.0	22.8	1.54 (0.51)	1.52 (0.52)	22.9	19.9
	−2	−7	1.51 (0.17)	1.64 (0.21)	94.4	96.2	1.50 (0.17)	1.50 (0.19)	93.8	85.3	1.50 (0.18)	1.50 (0.19)	90.3	84.1
		−8	1.51 (0.28)	1.64 (0.34)	57.7	63.8	1.49 (0.28)	1.50 (0.20)	56.7	47.3	1.50 (0.30)	1.50 (0.31)	50.3	46.0
		−9	1.53 (0.47)	1.66 (0.56)	29.9	32.2	1.52 (0.46)	1.50 (0.49)	28.7	23.3	1.54 (0.51)	1.52 (0.52)	22.9	19.9
	−3	−7	1.51 (0.17)	1.71 (0.22)	94.6	97.8	1.50 (0.17)	1.50 (0.19)	94.2	84.7	1.50 (0.18)	1.50 (0.19)	90.3	84.3
		−8	1.51 (0.28)	1.71 (0.35)	58.4	69.6	1.50 (0.28)	1.50 (0.30)	56.8	47.6	1.50 (0.30)	1.50 (0.31)	50.3	46.0
		−9	1.54 (0.47)	1.73 (0.59)	30.3	35.4	1.53 (0.47)	1.50 (0.50)	28.7	23.2	1.54 (0.51)	1.52 (0.52)	22.9	19.9
	−4	−7	1.51 (0.17)	1.76 (0.23)	94.7	98.2	1.50 (0.17)	1.50 (0.19)	93.1	84.9	1.50 (0.18)	1.50 (0.19)	90.3	84.4
		−8	1.51 (0.28)	1.75 (0.37)	58.7	72.2	1.50 (0.29)	1.50 (0.31)	56.4	46.4	1.50 (0.30)	1.50 (0.31)	50.3	46.0
		−9	1.54 (0.48)	1.77 (0.61)	30.6	37.2	1.53 (0.48)	1.50 (0.50)	27.5	22.2	1.54 (0.51)	1.52 (0.52)	22.9	19.9
2.00	0	−7	1.98 (0.19)	2.01 (0.22)	100.0	100.0	1.98 (0.19)	2.01 (0.22)	100.0	100.0	1.99 (0.22)	2.02 (0.23)	100.0	100.0
		−8	1.99 (0.32)	2.00 (0.36)	97.3	93.7	1.99 (0.32)	2.01 (0.36)	97.3	93.6	2.00 (0.36)	2.01 (0.38)	94.8	92.7
		−9	2.03 (0.54)	2.01 (0.60)	70.0	59.0	2.02 (0.55)	2.02 (0.61)	68.9	58.5	2.05 (0.59)	2.03 (0.63)	62.2	56.4
	−2	−7	2.01 (0.20)	2.25 (0.25)	100.0	100.0	1.99 (0.20)	2.00 (0.22)	100.0	100.0	2.00 (0.22)	2.01 (0.23)	100.0	100.0
		−8	2.01 (0.33)	2.26 (0.42)	97.4	97.5	2.00 (0.32)	2.01 (0.37)	97.1	93.6	2.00 (0.36)	2.01 (0.38)	94.7	92.8
		−9	2.07 (0.56)	2.28 (0.71)	70.6	68.1	2.05 (0.55)	2.01 (0.60)	69.8	57.7	2.07 (0.61)	2.02 (0.64)	62.4	54.9
	−3	−7	2.03 (0.20)	2.38 (0.27)	100.0	100.0	1.99 (0.20)	2.01 (0.22)	100.0	100.0	2.00 (0.22)	2.01 (0.23)	100.0	100.0
		−8	2.02 (0.33)	2.38 (0.45)	97.3	98.6	1.99 (0.32)	2.00 (0.37)	96.9	93.5	2.00 (0.36)	2.01 (0.38)	94.5	92.5
		−9	2.06 (0.56)	2.41 (0.75)	70.9	72.2	2.04 (0.55)	2.01 (0.60)	69.7	57.2	2.06 (0.61)	2.03 (0.63)	62.3	55.0
	−4	−7	2.03 (0.20)	2.46 (0.28)	100.0	100.0	2.00 (0.20)	2.00 (0.23)	100.0	100.0	2.00 (0.22)	2.01 (0.23)	100.0	100.0
		−8	2.03 (0.33)	2.46 (0.47)	97.3	99.1	2.00 (0.33)	2.00 (0.37)	97.0	93.3	2.00 (0.36)	2.01 (0.38)	94.5	92.5
		−9	2.07 (0.56)	2.48 (0.78)	70.8	75.1	2.04 (0.56)	2.01 (0.61)	67.0	56.2	2.06 (0.61)	2.03 (0.55)	62.1	55.0
4.00	0	−7	3.99 (0.30)	4.00 (0.35)	100.0	100.0	3.99 (0.30)	4.00 (0.34)	100.0	100.0	3.99 (0.36)	4.01 (0.37)	100.0	100.0
		−8	3.99 (0.50)	4.01 (0.56)	100.0	100.0	3.99 (0.50)	4.01 (0.55)	100.0	100.0	4.01 (0.59)	4.02 (0.60)	100.0	100.0
		−9	4.02 (0.83)	4.03 (0.93)	100.0	100.0	4.02 (0.83)	4.03 (0.92)	100.0	100.0	4.07 (0.97)	4.07 (1.02)	99.6	99.8
	−2	−7	4.05 (0.31)	4.93 (0.46)	100.0	100.0	3.99 (0.31)	3.99 (0.35)	100.0	100.0	3.99 (0.36)	4.00 (0.37)	100.0	100.0
		−8	4.06 (0.51)	4.95 (0.73)	100.0	100.0	4.00 (0.50)	4.00 (0.55)	100.0	100.0	4.01 (0.59)	4.02 (0.60)	100.0	100.0
		−9	4.09 (0.85)	5.01 (1.24)	100.0	100.0	4.03 (0.83)	4.03 (0.92)	100.0	100.0	4.07 (0.97)	4.07 (1.02)	99.6	99.8
	−3	−7	4.08 (0.32)	5.46 (0.53)	100.0	100.0	3.99 (0.31)	3.99 (0.36)	100.0	100.0	3.99 (0.36)	4.00 (0.38)	100.0	100.0
		−8	4.09 (0.52)	5.48 (0.83)	100.0	100.0	4.00 (0.50)	4.00 (0.56)	100.0	100.0	4.01 (0.59)	4.02 (0.60)	100.0	100.0
		−9	4.12 (0.86)	5.55 (1.42)	100.0	100.0	4.03 (0.84)	4.03 (0.92)	100.0	100.0	4.07 (0.97)	4.07 (1.02)	99.6	99.8
	−4	−7	4.09 (0.32)	5.80 (0.57)	100.0	100.0	3.99 (0.33)	4.00 (0.39)	100.0	100.0	3.99 (0.36)	4.00 (0.38)	100.0	100.0
		−8	4.10 (0.52)	5.82 (0.92)	100.0	100.0	4.00 (0.51)	4.01 (0.58)	100.0	100.0	4.01 (0.59)	4.02 (0.60)	100.0	100.0
		−9	4.14 (0.87)	5.89 (1.54)	100.0	100.0	4.04 (0.85)	4.03 (0.94)	100.0	100.0	4.07 (0.97)	4.07 (1.02)	99.6	99.8

Abbreviations: SAE, serious adverse events; SCCS, self-controlled case series; SD, standard deviation.

^a^Control intervals included ${C}_0\left(14\ \mathrm{days}\right),{C}_1\left(14\ \mathrm{days}\right),\mathrm{and}$  ${C}_2\left(28\ \mathrm{days}\right)$ with a total person-time of 56 days among those who received dose 2; control intervals included ${C}_0\left(14\ \mathrm{days}\right)\ \mathrm{and}$  ${C}_1\left(49\ \mathrm{days}\right)$ with a total person-time of 63 days among those who did not receive dose 2.

^c^Propensity score approach for SCCS design.

^c^Partitioned SCCS approach.

^d^The risk interval after dose 1.

^e^The risk interval after dose 2.

When ${\mathrm{P}}_{V_2}^0=40\%$, ${\mathrm{\pi}}_1=-4$, ${\mathrm{\beta}}_0=-9$, and ${e}^{\beta_1}={e}^{\beta_2}=4.0$, standard SCCS gave ${e}^{{\hat{\mathrm{\beta}}}_2}=6.18$ for ${\mathrm{RI}}_2$, a 54.5% bias compared with the true value of 4.0, while estimates from both PS-SCCS and P-SCCS were only slightly biased (Table 3).

Similar findings were observed when ${\mathrm{P}}_{V_2}^0$increased to 80%, although the bias decreased, and empirical power was less inflated for ${e}^{{\hat{\mathrm{\beta}}}_2}$ (Table 4). PS-SCCS and P-SCCS still performed well.

We observed similar simulation results when utilizing 14-day risk intervals after doses 1 and 2 (Tables 5 and 6). Notably, the standard SCCS yielded a more biased RI after dose 2 with14-day risk intervals compared with 7-day risk intervals, while PS-SCCS and P-SCCS continued to yield either unbiased or slightly biased estimates.

**Table 5:** Under the alternative hypothesis (${\mathrm{\beta}}_1$=${\mathrm{\beta}}_2\ne 0$), a 14-day risk interval for doses 1 and 2 (${R}_1={R}_2=14\ \mathrm{days}$), and baseline rate of dose 2 vaccination = 40%, the estimated RIs (${e}^{{\hat{\mathrm{\beta}}}_1}$and ${e}^{{\hat{\mathrm{\beta}}}_2}$) (standard deviation) and empirical power by relative incidences (${\mathrm{\beta}}_1$ and ${\mathrm{\beta}}_2$), the impact of experiencing SAEs during ${R}_1$ on dose 2 vaccination (${\mathrm{\pi}}_1$), and baseline SAE rate (β_0_) from 1000 simulated datasets with 10 000 individuals for each dataset.

**Simulation parameters**			**Standard SCCS** ^**a**^				**PS-SCCS** ^**b**^				**P-SCCS** ^**c**^			
			${\boldsymbol{e}}^{{\hat{\mathrm{\beta}}}_{\mathbf{1}}}$ **(SD)**	${\boldsymbol{e}}^{{\hat{\mathrm{\beta}}}_{\mathbf{2}}}$ **(SD)**	**Empirical power, %**		${\boldsymbol{e}}^{{\hat{\mathrm{\beta}}}_{\mathbf{1}}}$ **(SD)**	${\boldsymbol{e}}^{{\hat{\mathrm{\beta}}}_{\mathbf{2}}}$ **(SD)**	**Empirical power, %**		${\boldsymbol{e}}^{{\hat{\mathrm{\beta}}}_{\mathbf{1}}}$ **(SD)**	${\boldsymbol{e}}^{{\hat{\mathrm{\beta}}}_{\mathbf{2}}}$ **(SD)**	**Empirical power, %**	
**(** ${\boldsymbol{e}}^{{\boldsymbol{\mathrm{\beta}}}_{\mathbf{1}}}$ **) = (** ${\boldsymbol{e}}^{{\boldsymbol{\mathrm{\beta}}}_{\mathbf{2}}}$ **)**	${\boldsymbol{\Pi}}_{\mathbf{1}}$	${\boldsymbol{\mathrm{\beta}}}_{\mathbf{0}}$	${\boldsymbol{R}}_{\mathbf{1}}$ ^**d**^	${\boldsymbol{R}}_{\mathbf{2}}$ ^**e**^	${\boldsymbol{R}}_{\mathbf{1}}$ ^**d**^	${\boldsymbol{R}}_{\mathbf{2}}$ ^**e**^	${\boldsymbol{R}}_{\mathbf{1}}$ ^**d**^	${\boldsymbol{R}}_{\mathbf{2}}$ ^**e**^	${\boldsymbol{R}}_{\mathbf{1}}$ ^**d**^	${\boldsymbol{R}}_{\mathbf{2}}$ ^**e**^	${\boldsymbol{R}}_{\mathbf{1}}$ ^**d**^	${\boldsymbol{R}}_{\mathbf{2}}$ ^**e**^	${\boldsymbol{R}}_{\mathbf{1}}$ ^**d**^	${\boldsymbol{R}}_{\mathbf{2}}$ ^**e**^
1.50	0	−7	1.50 (0.13)	1.50 (0.21)	99.6	78.4	1.50 (0.13)	1.50 (0.21)	99.6	79.8	1.51 (0.16)	1.50 (0.24)	97.0	71.8
		−8	1.51 (0.22)	1.52 (0.35)	79.0	43.8	1.51 (0.22)	1.51 (0.34)	79.0	43.9	1.52 (0.27)	1.53 (0.40)	65.9	35.0
		−9	1.54 (0.37)	1.56 (0.59)	42.9	21.1	1.54 (0.37)	1.55 (0.56)	42.7	20.7	1.58 (0.45)	1.63 (0.77)	30.6	14.8
	−2	−7	1.54 (0.14)	2.05 (0.32)	99.6	99.4	1.50 (0.13)	1.50 (0.22)	99.6	79.9	1.51 (0.16)	1.50 (0.24)	97.0	71.3
		−8	1.55 (0.23)	2.09 (0.52)	83.1	82.2	1.51 (0.22)	1.51 (0.34)	79.3	44.1	1.52 (0.27)	1.53 (0.40)	65.9	34.6
		−9	1.59 (0.38)	2.20 (0.93)	47.5	46.1	1.54 (0.38)	1.57 (0.61)	41.9	20.9	1.58 (0.45)	1.63 (0.77)	30.6	14.9
	−3	−7	1.55 (0.14)	2.19 (0.34)	99.6	99.5	1.50 (0.14)	1.50 (0.23)	99.4	77.6	1.51 (0.16)	1.50 (0.24)	97.0	71.5
		−8	1.56 (0.23)	2.23 (0.57)	83.5	86.0	1.51 (0.23)	1.51 (0.36)	78.2	42.8	1.52 (0.27)	1.53 (0.40)	65.9	34.9
		−9	1.59 (0.39)	2.34 (1.01)	48.4	50.9	1.55 (0.40)	1.60 (0.71)	37.8	19.1	1.58 (0.45)	1.63 (0.77)	30.6	14.9
	−4	−7	1.55 (0.14)	2.24 (0.35)	99.6	99.7	1.50 (0.14)	1.50 (0.26)	99.2	75.1	1.51 (0.16)	1.50 (0.24)	97.0	71.6
		−8	1.56 (0.23)	2.29 (0.59)	83.6	87.6	1.51 (0.24)	1.52 (0.37)	73.7	40.1	1.52 (0.27)	1.53 (0.40)	65.9	34.9
		−9	1.60 (0.39)	2.41 (1.05)	48.5	53.7	1.57 (0.43)	1.62 (0.74)	33.9	16.3	1.58 (0.45)	1.63 (0.77)	30.6	14.9
2.00	0	−7	2.00 (0.16)	2.00 (0.26)	100.0	99.9	2.00 (0.16)	2.00 (0.25)	100.0	100.0	2.01 (0.20)	2.01 (0.29)	100.0	99.7
		−8	2.01 (0.26)	2.01 (0.42)	99.9	88.6	2.01 (0.26)	2.00 (0.40)	99.9	89.7	2.03 (0.33)	2.03 (0.50)	99.4	81.6
		−9	2.06 (0.44)	2.07 (0.69)	88.9	54.9	2.06 (0.44)	2.06 (0.65)	88.9	55.3	2.10 (0.57)	2.18 (0.95)	76.1	40.8
	−2	−7	2.06 (0.17)	2.96 (0.42)	100.0	100.0	2.00 (0.16)	2.00 (0.26)	100.0	99.8	2.01 (0.20)	2.01 (0.30)	100.0	99.5
		−8	2.08 (0.28)	3.00 (0.70)	99.9	99.3	2.01 (0.27)	2.01 (0.41)	99.9	89.3	2.03 (0.33)	2.03 (0.50)	99.4	81.6
		−9	2.13 (0.47)	3.18 (1.23)	91.0	83.3	2.06 (0.45)	2.08 (0.70)	87.7	54.5	2.10 (0.57)	2.18 (0.94)	76.1	40.4
	−3	−7	2.07 (0.17)	3.22 (0.48)	100.0	100.0	2.00 (0.17)	2.00 (0.28)	100.0	99.7	2.01 (0.20)	2.01 (0.30)	100.0	99.5
		−8	2.09 (0.28)	3.26 (0.78)	99.9	99.8	2.01 (0.27)	2.01 (0.43)	99.9	88.6	2.03 (0.33)	2.03 (0.50)	99.4	81.5
		−9	2.14 (0.47)	3.46 (1.39)	91.5	86.4	2.07 (0.49)	2.12 (0.82)	83.4	50.2	2.10 (0.57)	2.18 (0.95)	76.1	40.4
	−4	−7	2.08 (0.17)	3.33 (0.50)	100.0	100.0	2.00 (0.18)	2.00 (0.33)	100.0	99.6	2.01 (0.20)	2.01 (0.30)	100.0	99.5
		−8	2.09 (0.28)	3.38 (0.83)	99.9	99.9	2.02 (0.30)	2.02 (0.45)	99.6	86.4	2.03 (0.33)	2.03 (0.50)	99.4	81.5
		−9	2.14 (0.47)	3.59 (1.46)	91.7	87.3	2.09 (0.54)	2.15 (0.88)	79.8	45.7	2.10 (0.57)	2.18 (0.95)	76.1	40.3
4.00	0	−7	3.99 (0.26)	3.99 (0.41)	100.0	100.0	3.99 (0.26)	3.99 (0.39)	100.0	100.0	4.01 (0.35)	4.01 (0.51)	100.0	100.0
		−8	4.02 (0.44)	4.01 (0.66)	100.0	100.0	4.02 (0.44)	4.01 (0.62)	100.0	100.0	4.05 (0.59)	4.08 (0.87)	100.0	100.0
		−9	4.08 (0.73)	4.13 (1.09)	100.0	99.7	4.08 (0.73)	4.09 (0.98)	100.0	99.7	4.17 (1.01)	4.37 (1.70)	100.0	98.6
	−2	−7	4.18 (0.27)	7.44 (0.93)	100.0	100.0	3.99 (0.26)	3.99 (0.42)	100.0	100.0	4.01 (0.35)	4.01 (0.52)	100.0	100.0
		−8	4.21 (0.47)	7.58 (1.54)	100.0	100.0	4.02 (0.44)	4.01 (0.64)	100.0	100.0	4.05 (0.59)	4.08 (0.88)	100.0	100.0
		−9	4.28 (0.78)	8.02 (2.69)	100.0	100.0	4.08 (0.74)	4.11 (1.02)	100.0	99.6	4.17 (1.01)	4.37 (1.70)	100.0	98.5
	−3	−7	4.21 (0.28)	8.66 (1.14)	100.0	100.0	3.99 (0.28)	3.99 (0.47)	100.0	100.0	4.01 (0.35)	4.01 (0.52)	100.0	100.0
		−8	4.24 (0.47)	8.85 (1.91)	100.0	100.0	4.02 (0.46)	4.01 (0.68)	100.0	100.0	4.05 (0.59)	4.07 (0.88)	100.0	100.0
		−9	4.31 (0.79)	9.37 (3.38)	100.0	100.0	4.09 (0.77)	4.15 (1.20)	100.0	99.4	4.17 (1.01)	4.37 (1.70)	100.0	98.5
	−4	−7	4.22 (0.28)	9.26 (1.25)	100.0	100.0	4.00 (0.31)	4.01 (0.64)	100.0	100.0	4.01 (0.35)	4.01 (0.52)	100.0	100.0
		−8	4.25 (0.47)	9.45 (2.09)	100.0	100.0	4.02 (0.48)	4.01 (0.79)	100.0	100.0	4.05 (0.59)	4.07 (0.88)	100.0	100.0
		−9	4.33 (0.79)	10.06 (3.71)	100.0	100.0	4.12 (0.88)	4.22 (1.40)	100.0	99.2	4.17 (1.01)	4.37 (1.70)	100.0	98.6

Abbreviations: RI, relative incidence; SAE, serious adverse events; SCCS, self-controlled case series; SD, standard deviation.

^a^Control intervals included ${C}_0\left(14\ \mathrm{days}\right),{C}_1\left(14\ \mathrm{days}\right),\mathrm{and}$  ${C}_2\left(28\ \mathrm{days}\right)$ with a total person-time of 56 days among those who received dose 2; control intervals included ${C}_0\left(14\ \mathrm{days}\right)\ \mathrm{and}$  ${C}_1\left(49\ \mathrm{days}\right)$ with a total person-time of 63 days among those who did not receive dose 2.

^c^Propensity score approach for SCCS design.

^c^Partitioned SCCS approach.

^d^The risk interval after dose 1.

^e^The risk interval after dose 2.

**Table 6:** Under the alternative hypothesis (${\mathrm{\beta}}_1$=${\mathrm{\beta}}_2\ne 0$), a 14-day risk interval for doses 1 and 2 (${R}_1={R}_2=14\ \mathrm{days}$), and baseline rate of dose 2 vaccination = 80%, the estimated RIs (${e}^{{\hat{\mathrm{\beta}}}_1}$and ${e}^{{\hat{\mathrm{\beta}}}_2}$) (standard deviation) and empirical power by relative incidences (${\mathrm{\beta}}_1$ and ${\mathrm{\beta}}_2$), the impact of experiencing SAEs during ${R}_1$ on dose 2 vaccination (${\mathrm{\pi}}_1$), and baseline SAE rate (β_0_) from 1000 simulated datasets with 10 000 individuals for each dataset

**Simulation parameters**			**Standard SCCS** ^**a**^				**PS-SCCS** ^**b**^				**P-SCCS** ^**c**^			
			${\boldsymbol{e}}^{{\hat{\boldsymbol{\mathrm{\beta}}}}_{\mathbf{1}}}$ **(SD)**	${\boldsymbol{e}}^{{\hat{\boldsymbol{\mathrm{\beta}}}}_{\mathbf{2}}}$ **(SD)**	**Empirical power, %**		${\boldsymbol{e}}^{{\hat{\boldsymbol{\mathrm{\beta}}}}_{\mathbf{1}}}$ **(SD)**	${\boldsymbol{e}}^{{\hat{\boldsymbol{\mathrm{\beta}}}}_{\mathbf{2}}}$ **(SD)**	**Empirical power, %**		${\boldsymbol{e}}^{{\hat{\boldsymbol{\mathrm{\beta}}}}_{\mathbf{1}}}$ **(SD)**	${\boldsymbol{e}}^{{\hat{\boldsymbol{\mathrm{\beta}}}}_{\mathbf{2}}}$ **(SD)**	**Empirical power, %**	
**(** ${\boldsymbol{e}}^{{\boldsymbol{\mathrm{\beta}}}_{\mathbf{1}}}$ **) = (** ${\boldsymbol{e}}^{{\boldsymbol{\mathrm{\beta}}}_{\mathbf{2}}}$ **)**	${\boldsymbol{\Pi}}_{\mathbf{1}}$	β_0_	${\boldsymbol{R}}_{\mathbf{1}}$ ^**d**^	${\boldsymbol{R}}_{\mathbf{2}}$ ^**e**^	${\boldsymbol{R}}_{\mathbf{1}}$ ^**d**^	${\boldsymbol{R}}_{\mathbf{2}}$ ^**e**^	${\boldsymbol{R}}_{\mathbf{1}}$ ^**d**^	${\boldsymbol{R}}_{\mathbf{2}}$ ^**e**^	${\boldsymbol{R}}_{\mathbf{1}}$ ^**d**^	${\boldsymbol{R}}_{\mathbf{2}}$ ^**e**^	${\boldsymbol{R}}_{\mathbf{1}}$ ^**d**^	${\boldsymbol{R}}_{\mathbf{2}}$ ^**e**^	${\boldsymbol{R}}_{\mathbf{1}}$ ^**d**^	${\boldsymbol{R}}_{\mathbf{2}}$ ^**e**^
1.50	0	−7	1.50 (0.13)	1.50 (0.24)	99.6	72.0	1.50 (0.13)	1.50 (0.23)	99.6	72.8	1.51 (0.16)	1.51 (0.27)	97.0	62.7
		−8	1.51 (0.22)	1.53 (0.40)	79.5	36.6	1.51 (0.22)	1.52 (0.38)	79.5	37.5	1.52 (0.27)	1.55 (0.46)	65.9	29.8
		−9	1.54 (0.36)	1.58 (0.68)	43.6	18.2	1.54 (0.36)	1.56 (0.64)	43.4	17.2	1.58 (0.45)	1.69 (0.93)	30.6	11.3
	−2	−7	1.53 (0.13)	2.08 (0.36)	99.6	97.9	1.50 (0.13)	1.50 (0.24)	99.6	71.6	1.51 (0.16)	1.51 (0.27)	97.0	62.3
		−8	1.54 (0.23)	2.13 (0.60)	82.4	73.8	1.51 (0.22)	1.52 (0.39)	79.5	37.4	1.52 (0.27)	1.55 (0.46)	65.9	29.7
		−9	1.58 (0.38)	2.26 (1.08)	46.7	39.4	1.55 (0.39)	1.61 (0.74)	40.7	16.6	1.58 (0.45)	1.69 (0.93)	30.6	11.3
	−3	−7	1.54 (0.14)	2.20 (0.38)	99.6	98.7	1.50 (0.14)	1.50 (0.26)	99.4	70.7	1.51 (0.16)	1.51 (0.27)	97.0	62.6
		−8	1.55 (0.23)	2.26 (0.65)	82.5	78.2	1.51 (0.23)	1.53 (0.41)	77.3	35.7	1.52 (0.27)	1.55 (0.46)	65.9	29.6
		−9	1.58 (0.38)	2.40 (1.18)	47.5	43.6	1.56 (0.41)	1.65 (0.87)	36.5	14.5	1.58 (0.45)	1.69 (0.93)	30.6	11.3
	−4	−7	1.54 (0.14)	2.25 (0.39)	99.6	99.2	1.50 (0.14)	1.51 (0.29)	98.9	67.8	1.51 (0.16)	1.51 (0.27)	97.0	62.3
		−8	1.55 (0.23)	2.31 (0.67)	82.6	80.7	1.52 (0.25)	1.54 (0.43)	72.0	34.0	1.52 (0.27)	1.55 (0.46)	65.9	29.6
		−9	1.58 (0.38)	2.46 (1.21)	47.8	45.3	1.57 (0.44)	1.68 (0.92)	33.0	12.6	1.58 (0.45)	1.69 (0.93)	30.6	11.3
2.00	0	−7	2.00 (0.16)	2.00 (0.28)	100.0	98.9	2.00 (0.16)	2.01 (0.27)	100.0	99.2	2.01 (0.20)	2.02 (0.33)	100.0	98.2
		−8	2.01 (0.26)	2.02 (0.47)	99.9	81.8	2.01 (0.26)	2.02 (0.44)	99.9	82.1	2.03 (0.33)	2.06 (0.56)	99.4	71.6
		−9	2.05 (0.44)	2.11 (0.80)	88.9	44.7	2.06 (0.44)	2.08 (0.73)	89.1	45.6	2.10 (0.57)	2.26 (1.18)	76.1	30.2
	−2	−7	2.05 (0.16)	3.01 (0.48)	100.0	100.0	2.00 (0.16)	2.01 (0.29)	100.0	99.1	2.01 (0.20)	2.02 (0.33)	100.0	98.1
		−8	2.06 (0.27)	3.07 (0.80)	99.9	98.5	2.01 (0.27)	2.02 (0.46)	99.9	81.8	2.03 (0.33)	2.06 (0.56)	99.4	71.5
		−9	2.11 (0.46)	3.28 (1.46)	90.9	76.4	2.06 (0.46)	2.12 (0.83)	86.7	44.2	2.10 (0.57)	2.26 (1.17)	76.1	30.0
	−3	−7	2.06 (0.17)	3.25 (0.52)	100.0	100.0	2.00 (0.17)	2.01 (0.31)	100.0	98.9	2.01 (0.20)	2.02 (0.33)	100.0	98.1
		−8	2.07 (0.28)	3.30 (0.88)	99.9	99.1	2.01 (0.27)	2.03 (0.48)	99.9	80.2	2.03 (0.33)	2.06 (0.56)	99.4	71.3
		−9	2.12 (0.46)	3.55 (1.63)	91.1	80.1	2.08 (0.51)	2.19 (1.04)	82.2	38.7	2.10 (0.57)	2.26 (1.17)	76.1	29.9
	−4	−7	2.06 (0.17)	3.35 (0.54)	100.0	100.0	2.00 (0.17)	2.02 (0.37)	100.0	98.2	2.01 (0.20)	2.02 (0.33)	100.0	98.0
		−8	2.07 (0.28)	3.41 (0.92)	99.9	99.3	2.02 (0.3)	2.04 (0.53)	99.5	75.8	2.03 (0.33)	2.06 (0.56)	99.4	71.3
		−9	2.12 (0.46)	3.67 (1.72)	91.2	81.9	2.10 (0.55)	2.23 (1.12)	78.9	34.2	2.10 (0.57)	2.26 (1.17)	76.1	29.9
4.00	0	−7	3.99 (0.26)	4.00 (0.46)	100.0	100.0	3.99 (0.25)	4.00 (0.42)	100.0	100.0	4.01 (0.35)	4.04 (0.58)	100.0	100.0
		−8	4.01 (0.43)	4.02 (0.73)	100.0	100.0	4.02 (0.43)	4.02 (0.67)	100.0	100.0	4.05 (0.59)	4.12 (0.99)	100.0	100.0
		−9	4.07 (0.73)	4.18 (1.26)	100.0	99.2	4.07 (0.73)	4.12 (1.11)	100.0	98.9	4.17 (1.01)	4.53 (2.15)	100.0	95.9
	−2	−7	4.14 (0.27)	7.62 (1.04)	100.0	100.0	3.99 (0.26)	4.00 (0.46)	100.0	100.0	4.01 (0.35)	4.04 (0.58)	100.0	100.0
		−8	4.17 (0.46)	7.80 (1.75)	100.0	100.0	4.02 (0.43)	4.02 (0.70)	100.0	100.0	4.05 (0.59)	4.12 (0.99)	100.0	100.0
		−9	4.23 (0.77)	8.34 (3.25)	100.0	100.0	4.08 (0.74)	4.15 (1.16)	100.0	98.8	4.17 (1.01)	4.54 (2.18)	100.0	95.8
	−3	−7	4.16 (0.27)	8.76 (1.26)	100.0	100.0	4.00 (0.28)	4.01 (0.55)	100.0	100.0	4.01 (0.35)	4.04 (0.58)	100.0	100.0
		−8	4.19 (0.46)	8.96 (2.12)	100.0	100.0	4.02 (0.45)	4.03 (0.75)	100.0	100.0	4.05 (0.59)	4.12 (0.99)	100.0	100.0
		−9	4.25 (0.77)	9.64 (4.04)	100.0	100.0	4.11 (0.82)	4.24 (1.57)	100.0	98.2	4.17 (1.01)	4.54 (2.18)	100.0	95.8
	−4	−7	4.17 (0.27)	9.30 (1.37)	100.0	100.0	4.00 (0.30)	4.02 (0.71)	100.0	100.0	4.01 (0.35)	4.04 (0.58)	100.0	100.0
		−8	4.20 (0.46)	9.51 (2.30)	100.0	100.0	4.01 (0.47)	4.04 (0.87)	100.0	100.0	4.05 (0.59)	4.12 (1.00)	100.0	100.0
		−9	4.26 (0.77)	10.28 (4.37)	100.0	100.0	4.12 (0.91)	4.39 (1.89)	100.0	96.6	4.17 (1.01)	4.54 (2.18)	100.0	95.8

Abbreviations: RI, relative incidence; SAE, serious adverse event; SCCS, self-controlled case series; SD, standard deviation.

^a^Control intervals included ${C}_0\left(14\ \mathrm{days}\right),{C}_1\left(14\ \mathrm{days}\right),\mathrm{and}$  ${C}_2\left(28\ \mathrm{days}\right)$ with a total person-time of 56 days among those who received dose 2; control intervals included ${C}_0\left(14\ \mathrm{days}\right)\ \mathrm{and}$  ${C}_1\left(49\ \mathrm{days}\right)$ with a total person-time of 63 days among those who did not receive dose 2.

^c^Propensity score approach for SCCS design.

^c^Partitioned SCCS approach.

^d^The risk interval after dose 1.

^e^The risk interval after dose 2.

### Example results: myocarditis/pericarditis after receiving 2-dose series of mRNA COVID-19 vaccine

For BNT162b2, where some individuals with myocarditis/pericarditis during ${R}_1$ received dose 2, we employed PS-SCCS. For mRNA-1273, where none of those who experienced myocarditis/pericarditis during ${R}_1$ received dose 2, the PS-SCCS approach was not applicable. Instead, we employed P-SCCS. For BNT162b2, there were 90 incident myocarditis/pericarditis cases without history of SARS-CoV-2 infection identified using *International Classification of Diseases*, *Tenth Revision*, codes (B33.22, B33.23, I30.^*^, I40.^*^, I31.9, and I51.4) during the observation period; 67 (74.4%) were confirmed by medical record review. We prespecified a risk interval of 14 days after receiving doses 1 and 2. An indicator for having myocarditis/pericarditis during ${R}_1$ was included in a propensity score model as a predictor for receiving dose 2 among BNT162b2 vaccinees. Stabilized weights were calculated for each individual in the cohort, and only the stabilized weights of myocarditis/pericarditis cases were used in the subsequent SCCS analyses. A prespecified 14-day healthy vaccination period before receiving dose 1 was excluded from the analyses. We also did not count the person-time after the first risk interval and before receiving dose 2 (${C}_1$) as a control interval.^8^ There were 29, 6, and 32 myocarditis/pericarditis cases in the control intervals, the risk interval after dose 1, and the risk interval after dose 2, respectively. RIs were 1.90 (95% confidence interval [CI], 0.79-4.60) and 12.92 (95% CI, 7.56-22.09) for doses 1 and 2, respectively, in the standard SCCS analysis. While PS-SCCS produced a similar RI for dose 1 (RI = 1.85 [95% CI, 0.75-4.59]), the RI for dose 2 was lower (RI = 11.05 [95% CI, 6.53-18.68]). In the P-SCCS analyses, to better estimate the baseline rate of the outcome, we used both ${C}_0$ and ${C}_2$ as the control intervals to estimate RIs separately, resulting in RI = 1.76 (95% CI, 0.72-4.28) for dose 1 and RI = 11.06 (95% CI, 6.58-18.57) for dose 2.

For mRNA-1273, 26 myocarditis/pericarditis events were confirmed and included in the final analyses. There were 13, 3, and 10 events in the control intervals, ${R}_1$, and ${R}_2$, respectively. The standard SCCS yielded an RI of 1.96 (95% CI, 0.56-6.91) after dose 1 and an RI of 7.87 (95% CI, 3.33-18.57) after dose 2. Since none of the three mRNA-1273 recipients with an event during ${R}_1$ received dose 2, and the PS-SCCS approach was not applicable, we used the P-SCCS approach, which yielded a similar RI of 1.82 (95% CI, 0.52-6.41) after dose 1, but a lower RI of 6.48 (95% CI, 2.83-14.83) after dose 2.

## Discussion

We developed PS-SCCS approach considering the influence of having adverse events during ${R}_1$ on receiving dose 2 in evaluating 2-dose primary series of mRNA COVID-19 vaccines. Simulation studies under 144 scenarios showed that, compared with standard SCCS, PS-SCCS produced unbiased RIs, proper type I error rates, and proper empirical statistical power. In assessing risk of myocarditis/pericarditis after receiving BNT162b2 among members aged 12-39 years with a risk interval of 14 days after doses 1 and 2, standard SCCS and PS-SCCS yielded similar RIs after dose 1 and different RIs after dose 2, consistent with simulation results. While the real-world example demonstrated only modest bias when employing standard SCCS, it is important to note that this is just one example and should not be extrapolated to make a general observation that only mild/moderate bias would arise when the assumption that vaccine-induced adverse events during ${R}_1$ had no impact on receiving dose 2 is violated. In addition, we observed that there was a greater bias with standard SCCS when using 14-day risk interval in the simulation. Our analyses using these novel approaches showed that both BNT162b2 and mRNA-1273 were associated with elevated risk of myocarditis/pericarditis within 14 days after dose 2 among individuals aged 12-39 years, with RIs of 11.05 (95% CI, 6.53-18.68) and 6.48 (95% CI, 2.83-14.83), respectively.

The PS-SCCS approach requires a cohort population, and the propensity score model cannot be derived from SCCS sample as it depends on future SAEs (after dose 2) to define the population for estimating the probability of receiving dose 2. For this study, we used a logistic regression model to obtain the two parameters in the propensity score model. It is worth noting that these parameters have closed-form solutions that are functions of the proportions of individuals who received dose 2 among those with and without SAEs during ${R}_1$. Additionally, to calculate stabilized weights, we require the marginal proportion of individuals who received dose 2 in the cohort. Therefore, we can obtain stabilized weights either with the logistic regression model or the closed-form solutions. Note that we only included an indicator for having myocarditis/pericarditis during ${R}_1$ in the propensity score model. Our simulation results showed that including time-invariant predictors in the propensity score model is unnecessary, as the SCCS design inherently accounts for time-invariant confounding factors (details not presented).

P-SCCS offers a solution to the problem of violating the assumption that a prior vaccine-associated SAE has no impact on subsequent vaccination. This is a simple, straightforward approach with modest loss of efficiency because it does not use all information to simultaneously estimate RIs after doses 1 and 2. P-SCCS is a viable alternative when PS-SCCS is not applicable in situations when none of those who experienced SAEs during ${R}_1$ received dose 2.

Although the focus of this work was on the 2-dose mRNA vaccine primary series, these two approaches could also prove helpful in evaluating other 2-dose vaccinations (eg, recombinant zoster vaccine) and may even have potential for application in multidose vaccine regimens.

There are several limitations. First, in the simulation study, for simplicity, we did not include time-varying covariates (eg, seasonality). However, we expect similar results if time-varying covariates were included in the simulation study. Second, we did not consider factors associated with receiving dose 1. In the simulation study and the real-world example, we started with those who had received dose 1. However, if there is a healthy vaccination period before dose 1 or dose 2, one can exclude these periods from these SCCS analyses as we did in assessing myocarditis/pericarditis risk after 2-dose vaccination of COVID-19 mRNA vaccines. Third, we did not consider the dependence of recurrent SAEs, for example, if a person with a prior SAE is prone to experience more episodes of the same kind of SAE. Testing for dependence of recurrent SAEs and a statistical model for handling the dependence are available in Farrington and Hocine,^22^ and Di Bartolomeo et al.^23^ In the real-world example, myocarditis/pericarditis was rare and was unlikely to reoccur in the observation period. Fourth, in analyzing real-world data, risk during the risk intervals might fluctuate, even though we assumed a constant risk level. Fifth, the risk might not have fully returned to the baseline level before transitioning from the risk interval to the control interval. A large sample size is needed to define the dynamic nature of the risk pattern throughout the observation period. Last, if individuals experienced mild adverse events that did not require medical attention, their medical conditions might not be captured in electronic health care records. The potential impact of these mild adverse events on subsequent vaccinations was not considered in our proposed approaches, which focused on SAE.

The newly proposed PS-SCCS and P-SCCS methods have been shown to produce unbiased RIs, proper type I error rates, and proper empirical statistical power by considering influence of vaccine-associated SAEs during ${R}_1$ on receiving dose 2. In addition, these approaches can accommodate recurrent SAEs after doses 1 and 2. Unlike the modified SCCS, which uses a pseudolikelihood approach and has a software package that is exclusively accessible in R, both PS-SCCS and P-SCCS are more familiar to statisticians and can be easily implemented in statistical software such as SAS as well. Through the application of these innovative methodologies, we have demonstrated an elevated risk of myocarditis/pericarditis within 14 days following the administration of dose 2 for both mRNA vaccines among individuals aged 12-39 years.

## Supplementary Material

Web_Material_kwae141

## Data Availability

Individual-level data reported in this study involving human research participants are not publicly shared due to potentially identifying or sensitive patient information. Upon request to the corresponding author, and subject to review and approval of an analysis proposal, KPSC may provide the deidentified aggregate-level data that support the findings of this study within 6 months. Anonymized data (deidentified data including participant data as applicable) that support the findings of this study may be made available from the investigative team in the following conditions: (1) agreement to collaborate with the study team on all publications, (2) provision of external funding for administrative and investigator time necessary for this collaboration, (3) demonstration that the external investigative team is qualified and has documented evidence of training for human subjects protections, and (4) agreement to abide by the terms outlined in data use agreements between institutions.
