# Supplementary material for: A propensity score approach and a partitioned approach for the self-controlled case series design to evaluate safety of a 2-dose vaccine series: application to myocarditis/pericarditis following mRNA COVID-19 vaccination
Source: Am J Epidemiol. 2024 Jun 21;194(1):254–66. doi: 10.1093/aje/kwae141 (PMC11735979; doi:10.1093/aje/kwae141)
Supplement: Web_Material_kwae141 [file web_material_kwae141.pdf]

Supplementary Material

A Propensity Score Approach and a Partitioned Approach for the Self-Controlled Case Series Design to Evaluate Safety of a 2-dose Vaccine Series: Application to myocarditis/pericarditis following mRNA COVID-19 vaccination

Stanley Xu, PhD[1,2], Lina S. Sy, MPH[1], Bing Han, PhD[1], Vennis Hong, MPH[1], Katia J. Bruxvoort, PhD[1,3], Bruno Lewin MD[1,4], Kimberly J. Holmquist, MPH[1], Lei Qian, PhD[1]

[1]Department of Research & Evaluation, Kaiser Permanente Southern California, 100 S. Los Robles Ave, 5th Floor, Pasadena, CA 91101

[2]Department of Health Systems Science, Kaiser Permanente Bernard J. Tyson School of Medicine, Pasadena, CA

[3]School of Public Health, University of Alabama at Birmingham, Birmingham, Al 35233

[4]Department of Clinical Science, Kaiser Permanente Bernard J. Tyson School of Medicine, Pasadena, CA

Appendix S1

Appendix S1. SAS codes for fitting three SCCS models

```
/*************************************************************
*  Title:      SAS codes for fitting three SCCS models
*              for 2-dose series such as COVID-19 mRNA vaccines
*  Developer:  Stanley Xu, PhD
*              Research and Evaluation
*              Kaiser Permanente Southern California
*              Email: stan.xu@kp.org
*  Date Created: 04/25/2024
*************************************************************/

libname mydir 'S:\R&E Scientists\Stanley Xu\';

/**********************************************************;
*Step 1: calculate stabilized weights (sw) using cohort data.
*in the cohort data, there are following variables:
*v2, an indicator for receiving the 2nd dose during observation period
*yr1, an indicator for having AE in risk interval 1
*case, an indicator for AE during observation period.
*two approaches are available for calculating sw
**********************************************************/

*first approach to calculate sw using logistic regression;
proc logistic data=mydir.cohort_data;
 model v2 (event='1')=yr1;
 output out=PS_2levels p=p_v2;
run;

ods output onewayfreqs=frq_tx;
proc freq data=mydir.cohort_data;
 tables v2;
```

```
run;

data _null_;
    set frq_tx;
    percent = percent/100;
    if v2=0 then call symputx('unvac', percent);
    if v2=1 then call symputx('seconddose', percent);
run;

data sw;
keep studyid sw iptw x;
 set PS_2levels;
 if v2=1 then iptw=1/p_v2;
 else if v2=0 then iptw=1/(1-p_v2);
 if v2=1 then sw=&seconddose*iptw;
 else if v2=0 then sw=&unvac*iptw;
run;

***select cases;
data sw_cases;
 set sw;
 if case=1;
run;


**second approach to calculate sw using closed forms;
ods output onewayfreqs=frq_tx;
proc freq data=mydir.cohort_data;
 tables v2;
run;

data _null_;
    set frq_tx;
    percent = percent/100;
    if v2=0 then call symputx('unvac', percent);
    if v2=1 then call symputx('seconddose', percent);
run;

ods output CrossTabFreqs=frq_cross;
proc freq data=mydir.cohort_data;
 tables yr1*v2;
run;

data _null_;
 set frq_cross;
 percent = percent/100;
 if yr1=0 and v2=1 then call symputx('v2_yr1_0', rowpercent);
 if yr1=1 and v2=1 then call symputx('v2_yr1_1', rowpercent);
run;

proc iml;
 v2_yr1_0=&v2_yr1_0/100;
 v2_yr1_1=&v2_yr1_1/100;
 pi_0=log(v2_yr1_0/(1-v2_yr1_0));
 pi_1=log(v2_yr1_1/(1-v2_yr1_1))-pi_0;
 call symputx('pi_0', pi_0);
 call symputx('pi_1', pi_1);
```

```
quit;

data sw_cases;
 set mydir.cohort_data;
 if case=1;   ***select case only;
 p_v2=exp(&pi_0+yr1*&pi_1)/(1+(exp(&pi_0+yr1*&pi_1)));
 if v2=1 then iptw=1/p_v2;
 else if v2=0 then iptw=1/(1-p_v2);
 if v2=1 then sw=&seconddose*iptw;
 else if v2=0 then sw=&unvac*iptw;
run;

/*********************************************************
*Step 2: process case data with the following variables:
*studyid
*start: date when observation starts
*end: date when observation ends
*vacdt1: date for dose 1, vacdt2: date for dose 2
*admdate: event date
********************************************************/

%let rw1=14; **risk interval after dose 1;
%let rw2=14; **risk interval after dose 2;
%let inputdt=mydir.myocases;

data daydt_t1;
 set &inputdt;
   rw1=&rw1;
   rw2=&rw2;;
  do day=start to end;
   surv=1; **dummy variable for calculating person time;
   if vacdt2 ne . then do;
     *first control window;
      if day<vacdt1 then do;
       window='c0';
       x=0;
      end;
      **first risk window;
      else if day=<(vacdt1+rw1-1) then do;
       window='r1';
       x=1;
      end;
      *second control window;
      else if day<(vacdt2) then do;
        window='c1';
        x=0;
      end;
      **second risk window;
       else if day=<(vacdt2+rw2-1) then do;
        window='r2';
        x=2;
       end;
       else do;
        window='c2';
        x=0;
       end;
    end;
```

```
   if vacdt2=. then do;
      *first control window;
      if day<vacdt1 then do;
        window='c0';
        x=0;
      end;
      **first risk window;
      else if day=<(vacdt1+rw1-1) then do;
        window='r1';
        x=1;
      end;
      *second control window;
      else do;
        window='c1';
        x=0;
       end;
   end;
  if day=admdate then y=1;
   else y=0;
   output;
  end;
run;

**exclude healthy vaccination period;
data daydt_exc_hvp;
 set daydt_t1;
*exclude the healthy vaccination period -14 to -1 before vaccination;
 if -14=<days_from_dose1<0 then delete;
*exclude the healthy vaccination before dose 2;
 if vacdt2 ne . and window='c1' then delete;
run;

**************************************************************************;
*Step 3: Standard SCCS---Xu et al Journal of Data Science 8(2010), 349-360;
**************************************************************************;

PROC PHREG DATA=daydt_exc_hvp;
 class x(ref='0');
   MODEL surv * y (0) = x/TIES=BRESLOW RISKLIMITS ALPHA=0.05;
   strata studyid;
run;

*********************************************;
**Step 4a: Partitioned SCCS for risk interval 1;
*********************************************;

data psccs_r1;
 set daydt_exc_hvp;
 if window in ('c0','r1','c2');
run;

PROC PHREG DATA=psccs_r1;
 class x(ref='0');
   MODEL surv * y (0) = x/TIES=BRESLOW RISKLIMITS ALPHA=0.05;
   strata studyid;
run;
```

```
********************************************;
**Step 4b: Partitioned SCCS for risk interval 2;
********************************************;
data psccs_r2;
 set daydt_exc_hvp;
 if window in ('c0','r2','c2');
run;

PROC PHREG DATA=psccs_r2;
 class x(ref='0');
   MODEL surv * y (0) = x/TIES=BRESLOW RISKLIMITS ALPHA=0.05;
   strata studyid;
run;


*************************;
*Step 5: PS-SCCS approach;
*************************;
*Step 5a: calculate person time and # of events for control and risk
intervals;
proc means data=daydt_exc_hvp sum;
 var surv y;
 class studyid x;
 ods output summary=AE_pt_individual;
run;

proc sort data=AE_pt_individual;
 by studyid;

data mrnonly;
 set AE_pt_individual;
 by studyid;
 if first.studyid;
run;

data empt3obs;
 keep studyid x;
 set mrnonly;
 do x=0 to 2;
  output;
 end;
run;

proc sort data=empt3obs;
 by studyid x;

proc sort data=AE_pt_individual;
 by studyid x;

data AE_pt_individual1;
 merge AE_pt_individual empt3obs;
 by studyid x;
run;

proc sort data=AE_pt_individual1;
 by studyid x;
```

```
data AE_pt_individual2;
   array yary[3] y_0 y_r1 y_r2;
   array ptary[3] pt_0 pt_r1 pt_r2;
   do ii=1 to 3 until(last.studyid);
    set AE_pt_individual1;
      by studyid;
      yary[ii]=y_Sum;
    ptary[ii]=surv_Sum;
   end;
run;


*Step 5b: merge with the dataset that has stablized weights;
proc sort data=mydir.myocases;
by studyid;

proc sort data=mydir.sw_cases;
by studyid;

proc sort data=AE_pt_individual2;
by studyid;

data AE_pt_individual3;
 merge AE_pt_individual2 myo.pfizer_mydir.sw_cases mydir.myocases;
 by studyid;
run;

*Step 5c: use NLMIXED to obtain MLE after adjusting for sw;
proc nlmixed data=AE_pt_individual3;
parm b1=0.2 b2=0.2;
**for those who only received one dose;
  if  pt_r2=. then do;
       mu_0=pt_0/(pt_0+pt_r1*exp(b1));
       mu_r1=(pt_r1*exp(b1))/(pt_0+pt_r1*exp(b1));
      loglike=log(mu_0**(sw*y_0))+log(mu_r1**(sw*y_r1));
  end;
**for those who got 2 doses;
  else do;
       mu_0=pt_0/(pt_0+pt_r1*exp(b1)+pt_r2*exp(b2));
       mu_r1=(pt_r1*exp(b1))/(pt_0+pt_r1*exp(b1)+pt_r2*exp(b2));
       mu_r2=(pt_r2*exp(b2))/(pt_0+pt_r1*exp(b1)+pt_r2*exp(b2));
     loglike=log(mu_0**(sw*y_0))+log(mu_r1**(sw*y_r1))+log(mu_r2**(sw*y_r2));
  end;
   model y_r1~general (loglike);
   ods output ParameterEstimates=NLMIX_adj;
run;

data NLMIX_adj1;
 keep IR IR_lower IR_upper;
 set NLMIX_adj;
 IR=exp(Estimate);
 IR_lower=exp(lower);
 IR_upper=exp(upper);
run;

proc print data=NLMIX_adj1;
run;
```
